# Feeding and the Rhodopsin Family G-Protein Coupled Receptors in Nematodes and Arthropods

**DOI:** 10.3389/fendo.2012.00157

**Published:** 2012-12-18

**Authors:** João C.R. Cardoso, Rute C. Félix, Vera G. Fonseca, Deborah M. Power

**Affiliations:** ^1^Molecular Comparative Endocrinology, Centre of Marine Sciences, Universidade do AlgarveFaro, Portugal

**Keywords:** rhodopsin GPCR, feeding, conservation, evolution, invertebrates

## Abstract

In vertebrates, receptors of the rhodopsin G-protein coupled superfamily (GPCRs) play an important role in the regulation of feeding and energy homeostasis and are activated by peptide hormones produced in the brain-gut axis. These peptides regulate appetite and energy expenditure by promoting or inhibiting food intake. Sequence and function homologs of human GPCRs involved in feeding exist in the nematode roundworm, *Caenorhabditis elegans* (*C. elegans*), and the arthropod fruit fly, *Drosophila melanogaster* (*D. melanogaster*), suggesting that the mechanisms that regulate food intake emerged early and have been conserved during metazoan radiation. Nematodes and arthropods are the most diverse and successful animal phyla on Earth. They can survive in a vast diversity of environments and have acquired distinct life styles and feeding strategies. The aim of the present review is to investigate if this diversity has affected the evolution of invertebrate GPCRs. Homologs of the *C. elegans* and *D. melanogaster* rhodopsin receptors were characterized in the genome of other nematodes and arthropods and receptor evolution compared. With the exception of bombesin receptors (BBR) that are absent from nematodes, a similar gene complement was found. In arthropods, rhodopsin GPCR evolution is characterized by species-specific gene duplications and deletions and in nematodes by gene expansions in species with a free-living stage and gene deletions in representatives of obligate parasitic taxa. Based upon variation in GPCR gene number and potentially divergent functions within phyla we hypothesize that life style and feeding diversity practiced by nematodes and arthropods was one factor that contributed to rhodopsin GPCR gene evolution. Understanding how the regulation of food intake has evolved in invertebrates will contribute to the development of novel drugs to control nematodes and arthropods and the pests and diseases that use them as vectors.

## Introduction

**Figure 1:**
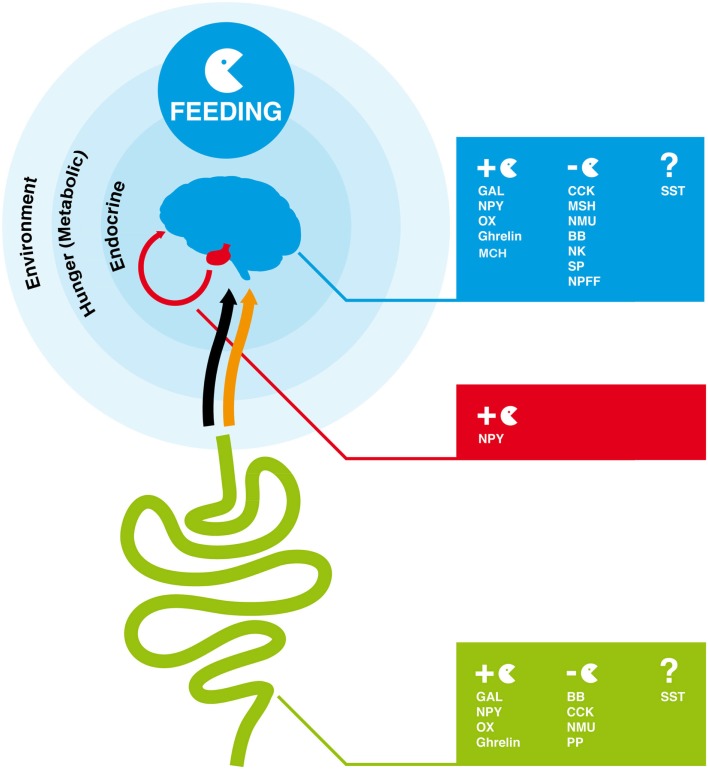
**Overview of endocrine factors that regulate feeding behavior in the human brain-gut axis**. In humans and other vertebrates, feeding is regulated by signals from the environment (odor and taste), hunger (metabolic signals), and endocrine signals produced by the gut and brain. The orange arrow represents the blood connection between gut and brain and the black arrow the nervous connection via the vagal afferent terminals through which peptides produced by the gut modulate the feeding response in the brain. GAL, NPY, OX, Ghrelin, and MCH are orexigenic peptides and promote appetite and feeding. CCK, MSH, NMU, BB, NK, SP, NPFF are anorexigenic. The role of SST peptides in feeding is unclear. The full peptide names are indicated in Table 1.

Feeding is the process by which food is obtained to provide energy. It must satisfy growth, survival, and reproductive requirements and has driven the evolution of specialized feeding behaviors and apparatus in metazoan. Regulation of feeding is a complex mechanism, which involves a combination of physical, chemical, and nutritional factors (Neary et al., 2004; Coll et al., 2007; Woods et al., 2008). Food-taking behavior is dependent on environmental signals (odors and taste), hunger signals (metabolic signals), and also endocrine satiety signals that via the blood stream or the vagal afferent terminals act on the hypothalamus, brain stem, or afferent autonomic nerves to modulate feeding response (Figure 1; Konturek et al., 2004; Stanley et al., 2005; Chaudhri et al., 2006; Woods et al., 2006, 2008). In mammals, psychological factors such as mood (emotions) and food reward have also been shown to affect eating behavior (Christensen, 1993; Berridge, 1996). In vertebrates, a group of small regulatory peptides that are produced by the brain-gut axis play a major role in the endocrine regulation of feeding and control of energy homeostasis (Figure 1; Coll et al., 2007; Chaudhri et al., 2008). These peptide hormones are divided into two groups, those that stimulate appetite (orexigenic peptides) and induce food intake and those that cause loss of appetite (anorexigenic peptides) and reduce food consumption and increase energy expenditure (Ahima and Osei, 2001; Wilding, 2002; Suzuki et al., 2010). The action of such peptides involves the activation of specific G-protein coupled receptors (GPCRs), which undergo conformational changes and promote the activation of intracellular signaling mechanisms that ultimately lead to a cellular response (Table 1; Marinissen and Gutkind, 2001; Xu et al., 2004; Fredriksson and Schioth, 2005).

**Table 1:** **Rhodopsin GPCR family members and activating peptides that regulate food intake in mammals**.

Receptor	Subfamily	Members	Activating peptides	Effect on feed
α-Group	Melanocortin (MCR)	MC1R to 5R	Melanocortin peptides (MSH, ACTH, LPH)	Reduce
β-Group	Gastrin-cholecystokinin (CCKR)	CCK1R, 2R	Cholecystokinin (CCK), Gastrin	Reduce
	Neurokinin (NKR)	NK1R to 3R	Substance P (SP), substance K (SK), neuromedin K (NK)	Reduce
	Neuropeptide FF (NPFFR)	NPFF1R, 2R	Neuropeptide FF (NPFF), neuropeptide AF (NPAF)	Reduce
	Orexin (OXR)	OX1R, 2R	Orexin-A and B (OXA, B)	Stimulate
	Neuropeptide Y (NPYR)	NPYRY1 to 6	Neuropeptide Y (NPY), peptide YY (PYY), pancreatic polypeptide (PP)	Stimulate/reduce
	Bombesin (BBR)	BB1R to 3R	bombesin (BB), gastrin-releasing peptide (GRP), neuromedin C and B	Reduce
	Ghrelin/obestatin (GHSR/GPR39)	GHSR, GPR39	Ghrelin (GHS), obestatin	Stimulate/reduce
	Neuromedin U (NMUR)	NMU1R, 2R	Neuromedin U (NMU) and S (NMS)	Reduce
γ-Group	Somatostatin (SSTR)	SST1R to 5R	Somatostatin (SST)	Not clear
	Galanin (GALR)	GAL1R to 3R	Galanin (GAL), galanin-like peptide (GALP)	Stimulate
	Melanin concentrating hormone (MCHR)	MCH1R, 2R	Melanin concentrating hormone (MHC)	Stimulate

*Receptor subfamily members, activating peptides and their effect on feed (stimulation or reduction) are indicated. For references please consult the text*.

The involvement of GPCRs in the regulation of vertebrate feeding and appetite is well recognized (Shioda et al., 2008). Much less is known about their homologs and cognate activating peptides in non-vertebrates. However, comparative sequence approaches and functional studies suggest that the involvement of GPCRs in metazoa feeding behavior emerged early and has been maintained during the species radiation (Brody and Cravchik, 2000; Hewes and Taghert, 2001; Fredriksson and Schioth, 2005; Teng et al., 2008). GPCRs have emerged via gene or genome duplication events followed by selection of the gene duplicates. Understanding the origin of GPCRs represents a valuable tool for the characterization of basic physiological functions that have been maintained during evolution. The present review takes a comparative approach and targets rhodopsin GPCR subfamily members in the model species, *C. elegans* (a nematode) and *D. melanogaster* (a arthropod) that are sequence and function homologs of vertebrate GPCRs implicated in feeding regulation. To enrich the data and provide insight into how divergent life style and feeding strategies may have shaped receptor evolution in invertebrates the sequence of the target GPCRs were identified in other nematodes and arthropods with available genome data.

## The Vertebrate GPCRs Superfamily and Their Role in Feeding

**Figure 2:**
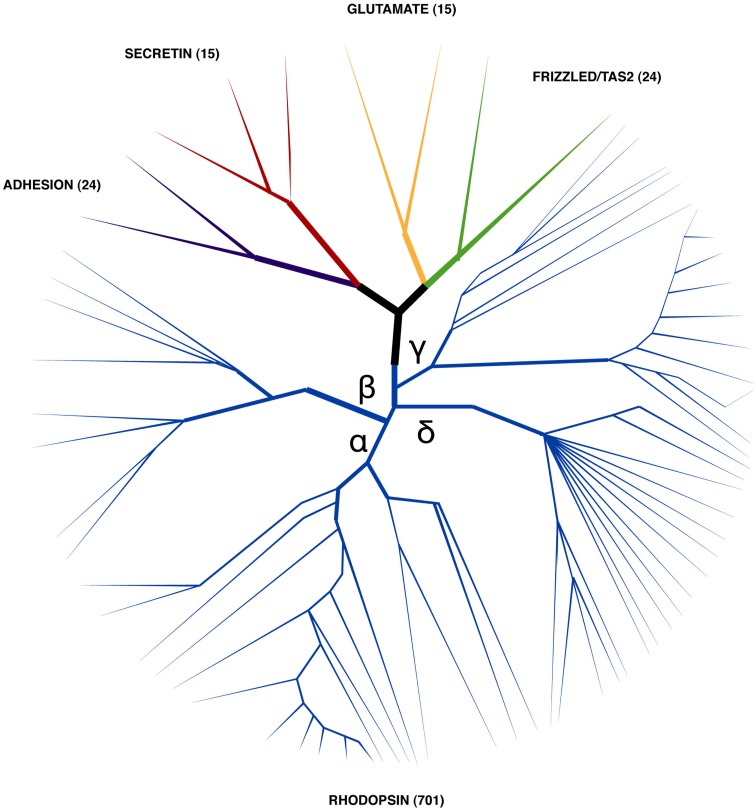
**Stylized phylogenetic tree showing the relationship between human GRAFS**. The number of human representatives identified within each superfamily is indicated within brackets (Fredriksson et al., 2003). Rhodopsin family members (which are represented by the blue branch) are the most numerous and their members are classified into four main sub-branches (α, β, γ, and δ). Human receptors, which are activated by peptides and have a role in feeding regulation, are members of the rhodopsin and secretin families.

G-protein coupled receptors are one of the largest groups of receptors present in cells. Based upon their structure and sequence similarity five distinct superfamilies have been defined in human: glutamate (G), Rhodopsin (R), Adhesion (A), Frizzled (F), and Secretin (S) and are collectively known as GRAFS (Fredriksson and Schioth, 2005; Figure 2). GPCRs are characterized by a signature motif of seven conserved transmembrane spanning helix domains (TM) in vertebrates and non-vertebrates. Receptor activation is mediated by the extracellular N-terminal domain and also by TM and extracellular loops (receptor core domain) that interact with diverse types of molecules. The cellular response is provoked by the receptor C-terminal domain which activates a series of intracellular signaling cascades via the G-protein coupled pathway complex (Bockaert and Pin, 1999; Marinissen and Gutkind, 2001). Other molecular mechanisms such assembly of receptor heterodimers and allosteric receptor–receptor interactions in the cell membrane are also involved in GPCR regulation, activation and signaling (Prinster et al., 2005; Langmead and Christopoulos, 2006; Fuxe et al., 2012).

G-protein coupled receptors are ubiquitous and involved in many different physiological functions. The glutamate receptors are involved in synaptic plasticity and participate in numerous functions in the central nervous system (CNS; Niswender and Conn, 2010). Rhodopsin receptors include receptors for hormones, neurotransmitters and photons and they are involved in taste, smell, and also regulate metabolism, reproduction, and neural function (Simoni et al., 1997; Murdoch and Finn, 2000; Gaillard et al., 2004; Waldhoer et al., 2004). Adhesion receptors participate in cell adhesion, signaling, and immune function (Bjarnadottir et al., 2007; Yona et al., 2008). Frizzled receptors are involved in the Wnt signaling pathway and in the control of cell proliferation and embryogenesis (van Amerongen and Nusse, 2009; Schulte, 2010). In contrast to other GPCRs, secretin family members are only activated by peptide hormones and they are implicated in brain-gut functions, calcium homeostasis, and in the stress response (McDermott and Kidd, 1987; Harmar, 2001; Bale and Vale, 2004; Moody et al., 2011). Due to their conserved structure and presence in many phyla, GPCRs are suggested to have a common evolutionary origin and to have arisen via gene/genome duplication early in the species radiation (Krishnan et al., 2012). With the exception of the glutamate family members, they are proposed to share a common ancestor with the cAMP receptors of primitive eukaryote species (Nordstrom et al., 2011).

In humans, more than 700 GPCR genes are predicted and a large proportion are orphans with unknown function (Figure 2). The rhodopsin family (a.k.a family A or class 1 GPCRs) comprise the most diverse receptor group and in humans they account for more than 80% of GPCRs and include members that are involved in regulation of feeding (Joost and Methner, 2002; Fredriksson et al., 2003). Rhodopsin family members possess a short N-terminal domain and are characterized by the presence of several conserved amino acid motifs such as N-S-x-x-N-P-x-x-Y within TM7 and the DRY (D(E)-R-Y(F)) motif between TM3 and intracellular loop (IL) 2 (Schioth and Fredriksson, 2005; Suwa et al., 2011). Based upon sequence similarity the human rhodopsin receptors are sub classified into four main groups (α, β, γ and δ; Figure 2; Fredriksson et al., 2003). The α-group contains clusters for the prostaglandin, amine, opsin, melatonin, melanocortin, endothelial, cannabinoid, and adenosine binding receptors. Members of the β-group include a subfamily of receptors for which known ligands are peptides such as orexin (OX), neuropeptide FF (NPFF), neurokinin (NK), gastrin-cholecystokinin (CCK), neuropeptide Y (NPY), endothelin-related (EDN), bombesin and related peptides (BB), neurotensin (NTS), ghrelin and obstatin, neuromedin (NMU), thyrotropin releasing hormone (TRH), arginine vasopressin (AVP), gonadotropin-releasing hormone (GNRH), and oxytocin (OXT). The γ group includes receptors for somatostatin (SST), opioids, galanin (GAL), melanin concentrating hormone (MCH), and chemokine peptides. The δ group contains the olfactory receptors (highly diverse > 400 members) as well as the glycoprotein, purine, and the MAS-related receptor clusters. In humans, twelve members of the rhodopsin family, which are activated by peptide hormones, play an important role in feed intake and stimulate or reduce food consumption (Table 1). The majority of these receptors are β group members and their role in the regulation of feed intake in mammals will now be briefly considered.

Receptors for melatonin (MT), gastrin-cholecystokinin (CCK), neurokinin (NK), neuropeptide FF (NPFF), bombesin and related peptides (BB), and neuromedin (NMU) have an inhibitory role in feed intake in vertebrates. Melanocortin receptors (MCR) are activated by melanocortin (ACTH, MSH, and lipotropin) peptides and administration of receptor agonists significantly reduces food consumption in rats (Irani and Haskell-Luevano, 2005). In addition mutant MC3R mice have increased fat mass (Coll et al., 2007) and ablation of the MC4R gene results in severe obesity (Coll et al., 2004; Millington, 2007). In rats, mutations of CCK1R are associated with obesity (Kopin et al., 1999) and peripheral administration of an NK1R antagonist leads to reduced weight gain after a high-fat diet (Karagiannides et al., 2011). Injection of NPFF provokes anorexia in mice and induces satiety (Murase et al., 1996; Bechtold and Luckman, 2006, 2007; Cline et al., 2009). BB peptides also mediate satiety (Hampton et al., 1998; Yamada et al., 2002; Gonzalez et al., 2008) and knockout BB2R mice have increased body weight (Ladenheim et al., 2002) and BB3R-deficient mice exhibit a mild obesity phenotype and increased food intake (Ohki-Hamazaki et al., 1997). Mice lacking the NMU gene are hyperphagic and have increased adiposity and obesity and amino acid variants in NMU are associated with human obesity (Brighton et al., 2004; Hainerova et al., 2006).

In contrast, orexin (OXs), neuropeptide Y (NPYs), galanin (GAL), and melanin concentrating hormone (MCH) receptors are activated by orexigenic peptides which stimulate feeding (Sakurai, 1999; Branchek et al., 2000; Chamorro et al., 2002; Lecklin et al., 2002; Lang et al., 2007; Wong et al., 2011). Administration of orexin-A and B stimulates food consumption in a dose-dependent manner (Sakurai et al., 1998; Matteri, 2001). NPY is one of the most potent orexigenic factors and NPY-induced feeding is markedly reduced in Y1-knockout mice and NPY Y1 receptor deficient mice lack appetite (Mercer et al., 2011; Pjetri et al., 2012). GAL1R-KO mice display increased food intake and body weight gain in response to an acute 3 day high-fat challenge (Zorrilla et al., 2007). MCH is a hypothalamic appetite-stimulating peptide that is high in obese mice (Kawauchi, 2006; Coll et al., 2007) and deletions in MCH1R confer resistance to diet-induced obesity (DIO) and MCH1R antagonists are effective in reducing body weight (Chung et al., 2011).

The role of SSTR and their activating peptides in vertebrates is unclear. In rats SSTR can stimulate or inhibit appetite although peptide injections in chickens have an orexigenic effect (Tachibana et al., 2009). In addition receptors for ghrelin-obestatin have opposing effects on feeding and ghrelin is associated with hunger scores and plasma ghrelin levels increase during fasting and decrease after food intake (Rocha-Sousa et al., 2010). Treatment of rats with obestatin suppresses food intake and decreases body weight gain (Zhang et al., 2005).

Other GPCR families activated by peptide hormones may also play a role in food intake and include members of the secretin receptor family: pituitary Adenylate-Cyclase Activating Peptide/Vasoactive Intestinal Peptide (PACR/VIPR; Morley et al., 1992; Chance et al., 1995); Glucagon and related peptide (GCGR/GLPR; McMahon and Wellman, 1997, 1998; Tang-Christensen et al., 2001; Woods et al., 2006); Calcitonin (CTR; Riediger et al., 2004) and Corticotrophin Releasing Factor (CRFR) receptors (Heinrichs and Richard, 1999; Bradbury et al., 2000; Richard et al., 2002). However, the secretin receptor family will not be considered in the present review.

## The Invertebrate GPCRs Superfamily

Invertebrates are one of the most diverse animal groups and they represent more than 95% of the species on Earth. Protostomia comprise the majority of the species identified and are of both ecological and economic importance as they are involved in the nutrient cycle, plant fertilization, and include agricultural pests and vectors of human disease, such as malaria and sleeping sickness. The divergence of Protostomes from Deuterostomes occurred more than 700 million years ago (MYA) and their success is associated with adaptations to a variety of ecological niches and modifications in their feeding habits that allow them to live, survive and reproduce in many different environments. Invertebrates can be herbivores (eating plant tissue, nectar, and pollen), carnivores (feeding on other invertebrates as well as larger animals), parasites (living on plant and animals), and detritus feeders (eating dead animal and plants). Surprisingly few studies exist about the regulation of feed intake in invertebrates, despite its importance for their success and this is also a neglected target for alternative control strategies. The genome of several invertebrates has been sequenced and in the metazoan Ensembl genome database (www.ensemblgenomes.org) 48 invertebrate genomes are available. Comparative molecular studies represent an invaluable mechanism to better understand invertebrate biology and to characterize endocrine factors associated with feeding.

Homologs of the vertebrate GPCR repertoire have been described in many invertebrates and representatives of the five distinct human GRAFS families are proposed to have emerged before the split of nematodes from the chordate lineage (Table 2; Fredriksson and Schioth, 2005). The model organisms, the nematode roundworm *C. elegans* and the fruit fly *D. melanogaster* are the most studied Prostostomes. Their genomes have been completely sequenced and are fully annotated and a vast range of functional resources exists and numerous GPCRs have been characterized (Consortium, 1998; Adams et al., 2000; Keating et al., 2003). In the roundworm, GPCRs account for approximately 5% of the genome (there are more than 1000) and the chemoreceptor genes, which are involved in chemoreception of environmental stimuli are unique in nematodes and are also the most abundant and diverse (Schioth and Fredriksson, 2005; Robertson and Thomas, 2006; Nagarathnam et al., 2012). In the fruit fly, approximately 200 GPCRs (1% of the genome) are predicted and the gustatory/taste receptors (Montell, 2009) are specific to insects although a quarter share sequence homology with vertebrate neurohormone receptors (Keating et al., 2003; Fredriksson and Schioth, 2005; Hauser et al., 2006; Nagarathnam et al., 2012). Recently GPCRs were also characterized in the genome of two Platyhelminthes, the blood fluke *Schistosoma mansoni* and the planarian *Schmidtea mediterranea* and a similar gene repertoire to vertebrates has been characterized. A platyhelminth-specific rhodopsin subfamily (PROF1) and a planarian-specific Adhesion-like family (PARF1) have been identified suggesting lineage specific GPCRs evolved in invertebrates (Suwa et al., 2011; Zamanian et al., 2011).

**Table 2:** **Gene number and receptor subfamilies of the human rhodopsin GPCRs involved in feeding and the sequence homologs identified in *C. elegans* and *D. melanogaster***.

Rhodopsin	Subfamily	Human	*C. elegans*	*D. melanogaster*
α-Group	Melanocortin	5	ni	ni
β-Group	Gastrin-cholecystokinin	2	2	2
	Neurokinin	3	6	5
	Neuropeptide FF	2		
	Orexin	2		
	Neuropeptide Y	6	12	4
	Bombesin	3	ni	2
	Ghrelin/obestatin	2	6	5
	Neuromedin U	2		
γ-Group	Somatostatin	5	6	2
	Galanin	3	3	2
	Melanin concentrating hormone	2	ni	ni
Total		37	35	22

*The total number of receptor genes in human, *C. elegans* and *D. melanogaster* is indicated. In *C. elegans* and *D. melanogaster* the homologs of the human Neurokinin/Neuropeptide FF/Orexin receptors and Ghrelin-Obestatin/Neuromedin U receptors were grouped due to their high sequence relatedness (Hewes and Taghert, 2001). ni, not identified*.

Comparison of the neuroendocrine GPCR complement in the fruit fly and the honey bee *Apis mellifera* (*A. mellifera*) revealed that a similar gene complement is present (Hauser et al., 2006). In the malaria vector, the mosquito *Anopheles gambiae* (*A. gambiae*) genome, a total of 276 GPCRs are predicted and approximately 30 correspond to putative neuropeptide receptors (Hill et al., 2002). With the exception of *C. elegans*, very little is known about GPCRs in other nematodes despite availability of molecular data in public databases. The activating molecules for the roundworm and fruit fly GPCRs in common with other organisms are in general neurohormones (biogenic amines, protein hormones, and neuropeptides) and they play a central role in the control of behavior, reproduction, development, feeding, and many other physiological processes. This suggests that GPCR signaling has been conserved during evolution and that neuropeptide signaling plays a key role in both Proto and Deuterostomes (Grimmelikhuijzen and Hauser, 2012).

The present review provides a general overview of the evolution of the rhodopsin GPCR members that are implicated in feeding regulation. It will start by identifying and describing sequence homologs of human rhodopsin GPCRs in the model invertebrate organisms *C. elegans* and *D. melanogaster* followed by the characterization of their homologs in other nematodes and arthropods with distinctive feeding habits and life styles (Table 4). The *C. elegans* and *D. melanogaster* rhodopsin GPCR repertoire was obtained from published data and to enrich and confirm the dataset it was complemented with appropriate database searches using the human homologs (Table 3). A total of 35 rhodopsin GPCRs are present in *C. elegans* and 22 in *D. melanogaster* genomes (Table 2) and a conserved role in feeding regulation has been demonstrated.

**Table 3:** **The human *C. elegans* and *D. melanogaster* rhodopsin GPCRs used for comparative sequence analysis and their accession numbers**.

Human		*C. elegans*		*D. melanogaster*	
Type	Accession number	Type	Accession number	Type	Accession number
BB1R	AAH95542.1	*ckr-1*	T23B3.4	AlCR2	CG13702
BB2R	AAA88050.1	*ckr-2*	Y39A3B.5	capaR	CG14575
BB3R	AAT79496.1	*nmur-1*	C48C5.1	CCHa1r	CG30106
CCK1R	NP_000721.1	*nmur-2*	K10B4.4	CCHa-2r	CG14593
CCK2R	NP_795344.1	*nmur-3*	F02E8.2A	CCKL-R17D3	CG32540
GALR1	NP_001471.2	*nmur-4*	C30F12.6	CCKL-R17D1	CG42301
GALR2	NP_003848.1	*npr-1*	C39E6.6	DAR-1	CG2872
GALR3	NP_003605.1	*npr-2*	T05A1.1A	DAR-2	CG10001
GHSR	AAI13548.1	*npr-3*	C10C6.2	DTKR	CG7887
GPR39	AAC26082.1	*npr-4*	C16D6.2	LKR	CG10626
MC1R	AAD41355.1	*npr-5*	Y58G8A.4	NepYr	CG5811
MC2R	NP_000520.1	*npr-6*	F41E7.3	NPFR1	CG1147
MCHR1	NP_005288.3	*npr-7*	F35G8.1	NKD	CG6515
NK1R	AAR23925.1	*npr-8*	C56G3.1B	PK-1R	CG9918
NK2R	AAH96842.1	*npr-9*	ZK455.3	PK-2-R2	CG8795
NK3R	AAR23926.1	*npr-10*	C53C7.1A	PK-2-R1	CG8784
NMUR-1	AAH51914.1	*npr-11*	C25G6.5	SNPFR	CG7395
NMUR-2	EAW61653.1	*npr-12*	T22D1.12	Star1-RA	CG7285
NPFF1R	NP_071429.1	*npr-13*	ZC412.1	CG10823	CG10823
NPFF2R	NP_004876.2	*npr-14*	W05B5.2	CG30340	CG30340
NPY1R	AAA59947.1	*npr-15*	T27D1.3	CG32547	CG32547
NPY2R	AAO92062.1	*npr-16*	F56B6.5	CG34381	CG34381
NPY4R	NP_005963.3	*npr-17*	C06G4.5		
NPY5R	NP_006165.1	*npr-18*	C43C3.2		
OX1R	AAC39601	*npr-20*	T07D4.1		
OX2R	AAC39602.1	*npr-21*	T23C6.5		
SSTR1	AAP84349.1	*npr-22*	Y59H11AL.1		
SSTR2	AAO92064.1	*npr-24*	R106.2		
SSTR3	AAP84354.1	*tkr-1*	C38C10.1		
SSTR4	AAS55648.1	*tkr-3*	AC7.1		
SSTR5	EAW85687.1	C49A9.7	C49A9.7		
		C50F7.1	C50F7.1		
		T02E9.1	T02E9.1		
		Y116A8B.5	Y116A8B.5		
		Y54E2A.1	Y54E2A.1		

## Feeding in Nematodes and Arthropods

Feeding in invertebrates in common with other animals involves a complex combination of physical, chemical, and nutritional factors (Chapman and De Boer, 1995). Taste and smell are important for feeding behavior and provide the CNS with information on quality and quantity of food and feeding behavior occurs mainly in response to both nutrient and nutritional storage status. Once feeding has been initiated and food ingested, the alimentary canal, and its associated glands triturate, lubricate, store, digest, and absorb the food material and excrete and expel unwanted remains (Audsley and Weaver, 2009).

The Nematoda is a highly diverse, complex, and specialized group of metazoans, about 30,000 species are currently known and many are renowned parasites (15%) and have specialized life cycles that depend on their host to survive and reproduce. Their success is associated with a protective, impermeable cuticle and by the diversity of the pharynx and feeding mechanisms (Coghlan, 2005). The shape and presence or absence of teeth, lancets, stylets, or other structures in the mouth reflects their distinct feeding methods. The majority of nematodes are free-living and inhabit soil and water and feed on microorganisms (bacteria, fungi, algae) and organic debris. The parasites feed on animal and plant tissues and some on vertebrate blood.

The Arthropoda represents the most diverse animal phyla and comprises over 80% of the species identified and the Insecta class is the most specious with approximately 920,000 species. Four main classes of feeding habits are recognized: plant feeders, predators (feed on aphids and mites), scavengers (feeding on dead and decaying organic matter), and parasites (of other insects and vertebrates), some of which are hematophagous. Within each of these classes, various types of feeding can be found such as biting and chewing on leaves or animal tissue and sucking from plant or animal cells or tissues. Despite this unique ability to use almost any organic substrate, most insect species restrict themselves to a particular category of food (Posnien et al., 2010) and feed primarily on a fluid diet (Prakash and Steele, 2010). The variety of feeding habits in arthropods is the result of anatomical and physiological adaptations to distinct food sources (Chapman and De Boer, 1995). The alimentary canal is composed of specialized regions that vary according to feeding habit and life stage.

The organisms selected for analysis of rhodospin GPCRs potentially involved in invertebrate feeding are members of different nematode and arthropod lineages. The specific life style and feeding habits of the invertebrates included in the analysis are indicated in Table 4.

**Table 4:** **Nematodes and arthropods used to analyze the rhodopsin GPCRs**.

	Life style	Feeding type	Databases
**NEMATODES**			
*Caenorhabditis elegans*	Free-living	Bacteria	http://metazoa.ensembl.org
			http://www.wormbase.org
*Caenorhabditis briggsae*	Free-living	Bacteria	http://metazoa.ensembl.org
*Caenorhabditis japonica*	Free-living	Bacteria, dead eggs and adult bugs	http://metazoa.ensembl.org
*Pristionchus pacificus* (necromenic nematode)	Parasitic	Bacteria, fungi and other nematodes	http://metazoa.ensembl.org
*Haemonchus contortus* (red stomach worm)	Parasitic	Bacteria, blood and tissue	http://www.sanger.ac.uk
*Brugia malayi* (filariasis worm)	Parasitic	Blood and lymphatic tissue	http://blast.ncbi.nlm.nih.gov
*Trichinella spiralis* (pork worm)	Parasitic	Mammalian cells and blood	http://metazoa.ensembl.org
*Meloidogyne incognita* (root-knot plant parasite)	Parasitic	Plant tissue	http://meloidogyne.toulouse.inra.fr
**ARTHROPODS**			
*Drosophila melanogaster* (fruit fly)	Free-living	Yeast	http://metazoa.ensembl.org
			http://www.flybase.org
*Apis mellifera* (honeybee)	Free-living	Nectar and pollen	http://metazoa.ensembl.org
*Bombyx mori* (silkworm)	Free-living	Plant leafs	http://metazoa.ensembl.org
*Aedes aegypti* (yellow fever mosquito)	Parasitic	Nectar and blood	http://metazoa.ensembl.org
*Anopheles gambiae* (malaria mosquito)	Parasitic	Nectar and blood	http://metazoa.ensembl.org
*Ixodes scapularis* (blacklegged tick)	Parasitic	Blood	http://metazoa.ensembl.org

*Information about life style, feeding type and the database interrogated is indicated*.

## Homologs of the Vertebrate Rhodopsin Family GPCRs Implicated in Feeding and Appetite Regulation in Non-Vertebrates

The following section describes the evolution and function of rhodopsin family members in nematodes and arthropods. It will start with an overview of those described in *C. elegans* and *D. melanogaster* involved in or candidates for feed intake regulation (Tables 2 and 5). Expression data when available from wormbase and flybase is included to provide insight into receptor function. It is followed by a section in which receptor evolution in invertebrates is discussed including homologs from non-model nematode and arthropod species.

**Table 5:** **An overview of the amino acid sequence similarity of the main subfamilies of *C. elegans* and *D. melanogaster* rhodopsin GPCRs and their human homologs**.

Rhodopsin	Subfamily	Characterized with function assigned		Novel members with an unknown role in feeding	
		*C. elegans* (%)	*D. melanogaster* (%)	*C. elegans* (%)	*D. melanogaster* (%)
α-Group	Gastrin-cholecystokinin	32–36	33–37		
β-Group	Neurokinin/neuropeptide FF/Orexin	38–43	27–45	30–38	27–33
	Neuropeptide Y	32–40	12–36	29–41	12–14
	Bombesin		38–40		
	Neuromedin U	35–41	29–41	30–36	21–25
γ-Group	Somatostatin		35–41	27–35	
	Galanin	36–40	37–43		

*Percentage of sequence similarity was calculated in the GeneDoc program (http://www.nr.bsc.org/gfx/genedoc/). The maximum and minimum sequence similarity of receptor subgroups between invertebrate and human homologs is indicated*.

**Figure 3:**
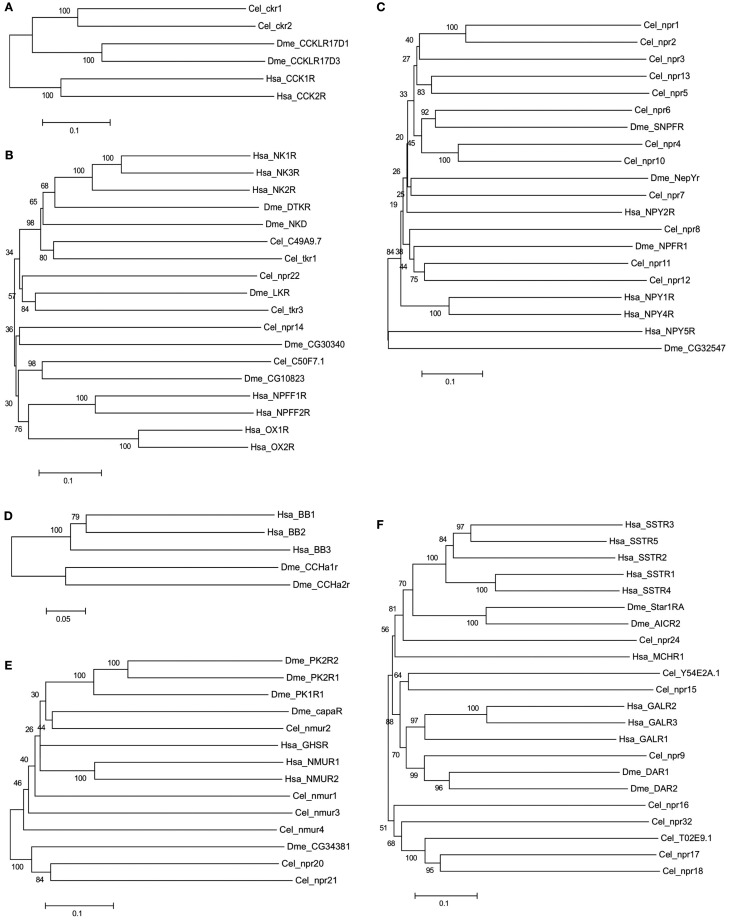
**Phylogenetic relationship of the Human (Hsa) rhodopsin GPCRs involved in feeding with the nematode *C. elegans* (Cel) and arthropod *D. melanogaster* (Dme) sequence homologs**. Trees were constructed using the neighbor joining method with 1000 bootstrap replicates (uniform rate among sites, pairwise deletion using the p-distance substitution model) built in the Mega5.1 program. Receptors were classified into six distinct subfamilies: **(A)** Gastrin-Cholecystokinin receptors; **(B)** Neurokinin/neuropeptide FF/orexin receptors, **(C)** Neuropeptide Y receptors, **(D)** Bombesin receptors, **(E)** Ghrelin/obstatin and Neuromedin U receptors, and **(F)** Somatostatin and galanin receptors. Accession numbers are described in Table 3.

In general, no putative melatonin peptide receptors (MCR) or melanin concentrating hormone receptor (MCHR) homologs have been described or were identified in the present study in any of the selected nematodes or arthropods (Figure 3). In addition, in nematodes no homolog of the vertebrate and fruit fly bombesin receptors seem to exist (Table 2). Duplicates of the human receptor genes were identified in the genomes of nearly all target species and phylogenetic analysis suggests specific gene duplication/deletions occurred within the nematode and arthropod lineages (Figure 3).

### The rhodopsin GPCRs in *C. elegans* and *D. melanogaster* genomes

#### Characterized and functionally assigned subfamily members

##### Gastrin-cholecystokinin receptor subfamily

In the genomes of *C. elegans* and *D. melanogaster* two putative Gastrin-CCK-like receptor homologs of the human members have been reported (Figure 3A; Keating et al., 2003; Janssen et al., 2008). In *C. elegans*, *ckr-1*, and *ckr-2* have been described and functionally characterized. The *ckr-1* is expressed in the nerve ring and functional RNAi knockdown studies reveal that loss of receptor activity provokes fat accumulation (McKay et al., 2007). However, if the receptors are ablated there is no apparent effect on feeding regulation but instead embryonic lethality and reduced brood size is observed (McKay et al., 2007). The neuropeptide *nlp-12* is the ligand of nematode *ckr-2* and the peptide receptor pair shares conserved biological activity with regards to fat storage with the human homolog (Janssen et al., 2008). A cognate peptide for nematode *ckr-1* is yet to be identified.

In *D. melanogaster* the two existent CCK-like receptors were designated CCKL-R17D3 (DSKR1) and CCKL-R17D1 (Kubiak et al., 2002). They are mainly expressed in the CNS and are activated by *Drosophila* sulfakinin (DSK; Nichols et al., 1988), which is a structurally and functionally related peptide to the vertebrate CCK (Audsley and Weaver, 2009). Their role in feeding regulation has not yet been demonstrated in *Drosophila* but in other arthropods the homolog receptor stimulation by SK causes gut emptying and satiety (Nichols, 2007). Injections of SK peptides significantly reduce meal size in locusts (*Schistocerca gregaria*; Wei et al., 2000) and cockroach (*Blattella germanica*; Maestro et al., 2001), carbohydrate feeding in the blowfly (*Phormia regina*), and inhibit female horse flies from blood feeding (Downer et al., 2007).

##### Neurokinin/neuropeptide FF/orexin receptor subfamily

In *C. elegans* two putative neurokinin (a.k.a. tachykinins) receptors *tkr-1* and *tkr-3* have been described (Keating et al., 2003; Greenwood et al., 2005). In *D. melanogaster* three neurokinin-like receptors have been reported: the neurokinin receptor (NKD), the tachykinin receptor (DTKR; Li et al., 1991; Monnier et al., 1992; Rosay et al., 1995; Poels et al., 2009), and the leucokinin receptor (LKR; Radford et al., 2002). Phylogenetic analysis of the invertebrate receptors suggests that they arose from an ancestral Neurokinin/neuropeptide FF/orexin-like receptor gene by species-specific duplication events prior to the Proto-Deuterostome divergence (Figure 3B; Hewes and Taghert, 2001). Characterization of the *C. elegans* *tkr-1* revealed expression is restricted to the socket cells (specialized nerve-accessory cells that act as an interface between the sensillum and hypodermis) and RNAi functional screens and the Nile Red fat assay revealed that this gene affects fat metabolism and fat droplet morphology and the pattern of fat deposition (Ashrafi et al., 2003). Knock down nematodes have a substantially lower fat content suggesting that this receptor is a key lipid storage regulator. *Tkr-3* RNAi studies caused mild sluggishness and slowed locomotion in nematodes (Keating et al., 2003), which may be related to modifications in the nervous system. *Tkr-3* is also present in the intestine but no role has yet been assigned in feeding and metabolism.

The *D. melanogaster* NKD and DTK receptors are expressed in the head of both larvae and adults and are activated by *Drosophila* tachykinin (DTK1–6) peptides, which are derived from the *drosotachykinin* (*Dtk*) gene (Birse et al., 2006; Poels et al., 2007) and also by substance P which is involved in the regulation of food intake and energy homeostasis in vertebrates (Birse et al., 2006; Poels et al., 2007). Knock down of DTKR in *D. melanogaster* modulated expression in both fed and starved flies of insulin-like peptides, which play a major role in the regulation of carbohydrates and lipid metabolism (Poels et al., 2009; Birse et al., 2011).

##### Neuropeptide Y receptor subfamily

In *C. elegans* four putative NPY-like receptors (*npr-1*, *npr-2, npr-5*, and *npr-11)* that share conserved sequence with the vertebrate NPYRs have been isolated and function characterized (de Bono and Bargmann, 1998; Keating et al., 2003; Kubiak et al., 2008; Cohen et al., 2009). Three NPY-like receptors have also been reported in *D. melanogaster*, these are the NepYr receptor and two neuropeptide F (NPF) receptors, the NPFR1 and the short NPFR (SNPFR; Figure 3C). The NPF peptide occurs as a long (NPF) and short (sNPF) isoform in arthropods (De Loof et al., 2001) and is the homolog of vertebrate neuropeptide Y (NPY; Li et al., 1992; de Jong-Brink et al., 2001).

In *C. elegans*, the nematode *npr-1* was the first receptor found to influence social feeding behavior and is predominantly expressed in the nervous system (de Bono and Bargmann, 1998). This receptor is activated by *flp-21* peptide (Rogers et al., 2003) and ablation of the peptide does not cause silencing of *npr-1* functions, suggesting that it can be activated by other molecules. In fact, *flp-18* peptide also activates *npr-1* and this peptide is also the ligand of *npr-5*, which is involved, in chemosensory response, foraging behavior, and fat metabolism (Rogers et al., 2003). Nematode *npr-5* is expressed in the head, neck, and body muscles and knock down and gene mutation studies revealed that in common with *npr-2* it is associated with intestinal fat storage regulation (Keating et al., 2003; Cohen et al., 2009), *dauer* formation, and other food-dependent decisions (Cohen et al., 2009). The *npr-11* has a role in reproduction and sensory dynamics of the olfactory system (Chalasani et al., 2010) but no role in feeding has yet been demonstrated (Chalasani et al., 2010).

The fruit fly NepYr and NPF receptors are expressed in the *D. melanogaster* CNS and NepYr is also present in the gut. NepYr is activated by dRYamide-1 and dRYamide-2, which has a C-terminal sequence similar to vertebrate NPY family peptides and in flies dRYamide suppresses feeding motivation (Ida et al., 2011). NPF and its receptors also modulate feeding behavior in *D. melanogaster* (Wu et al., 2003; Garczynski et al., 2005) and they promote feeding in larvae (Wu et al., 2003) and influence the effect of food deprivation in adult flies (Wu et al., 2003; Lingo et al., 2007). In other arthropods their functions have also been described and NPFR is involved in hindgut contraction in the bloodsucking bug (*Rhodnius prolixus*; Gonzalez and Orchard, 2009) and in ovarian maturation in locusts (Schoofs et al., 2001). In *D. melanogaster* sNPF is involved in the control of food intake and in the regulation of body size (Lee et al., 2004). Studies in mutant fruit flies over expressing sNPF peptide exhibit increased food intake and produce bigger and heavier flies, whereas sNPF loss-of-function mutants exhibit suppressed food intake (Lee et al., 2004). Gene expression studies with the red fire ant (*Solenopsis invicta Buren*) revealed SNPFR in brain is down-regulated during starvation (Chen and Pietrantonio, 2006) and expression of long NPF and its receptor in the malaria mosquito (*A. gambiae*) appear to be dependent on the insect nutritional status (Garczynski et al., 2005).

##### Bombesin receptor subfamily

Homologs of the vertebrate bombesin receptors have not been reported in nematodes and were not identified in the present study. Members of this family are only present in *D. melanogaster* and they correspond to the Allatostatin type B receptors (Stay, 2000). In *D. melanogaster*, two bombesin-like receptors have been isolated and function characterized: CCHamide-1r (CCHa1r; Johnson et al., 2003) and CCHamide-2r (CCHa-2r; Johnson et al., 2003; Hauser et al., 2008; Figure 3D).

In insects the function of the arthropod bombesin receptor is still poorly explored as a specific ligand has only recently been identified. CCHa-2r expression was detected in *D. melanogaster* brain and in the CNS and midgut of *B. mori* (Roller et al., 2008). Functional analysis reveals the receptors are activated by the peptides CCHamide-1 or CCHamide-2 that have been shown to suppress feeding activity in the cockroach, *Blattella germanica* (Audsley and Weaver, 2009).

##### Ghrelin-obestatin/neuromedin U receptor subfamily

In *C. elegans* four nmur-like receptors: *nmur-1*, *nmur-2*, *nmur-3*, and *nmur-4* have been described. In *D. melanogaster* the capaR and three pyrokinin receptors PK-1R, PK-2-R1, and PK-2-R2 are the homologs of vertebrate NMURs (Iversen et al., 2002; Park et al., 2002; Figure 3E). The nematode *nmur-1* is suggested to be involved in the sensory system and with processing information from specific food cues, which enables selection of different food types (Maier et al., 2010). *C. elegans* *nmur-2* was also shown with its ligand peptide (derived from the *nlp-44* precursor gene) to be involved in the regulation of food intake (Lindemans et al., 2009). To date no functional studies involving *nmur-3* and *nmur-4* have been reported although *nmur-4* is expressed in the pharynx and intestine suggesting it may have a role in feeding.

The *D. melanogaster* capaR is mainly expressed in the Malpighian tubules and it is involved in the increase of fluid transport and diuresis and no direct role in feeding has yet been attributed (Terhzaz et al., 2012). CapaR is activated by two neuropeptides, capa-1 and -2 that are encoded by the *capability* gene and have antidiurectic actions in insects (Pollock et al., 2004; Coast and Garside, 2005; Paluzzi et al., 2010). The *capability* gene also encodes the pyrokinin-1 (PK1) peptide that is a specific activator of PK-1R. PK-2-R1 and PK-2-R2 are activated by pyrokinin-2 (PK2) and Hug-γ that are derived from the hugin (hug) prepropeptide (Cazzamali et al., 2005).

Phylogenetic analysis of the pyrokinin receptors suggests that they share common ancestry and that PK-2-R1 and R2 are the result of a recent duplication in the fly genome. The pyrokinin peptides are involved in rhythmic motor activity in arthropods (Saideman et al., 2007) and receptors are expressed in the abdomen (carcass) and nervous tissue and involvement in modulation of feeding behavior has been suggested. Overexpression of the hugin gene was found to suppress feeding in *Drosophila*, while blockage of the synaptic activity of hugin neurons caused the opposite effect (Meng et al., 2002; Melcher and Pankratz, 2005).

##### Somatostatin receptor subfamily

A homolog of human SSTR in the *C. elegans* genome was predicted in the 1990’s (Wilson et al., 1994). Characterization of the deduced protein revealed that the signature motif of the vertebrate SSTR was missing in TM7, suggesting that the receptor is probably activated by other ligands. Since no other homolog of vertebrate SSTR has been reported, the function of the putative SSTR-like receptors in nematodes remains to be explored. In arthropods, Allatostatin type-C receptors are the homologs of the vertebrate somastostatin receptors and in *D. melanogaster*, two receptors star1-RA and AlCR2 were described (Kreienkamp et al., 2002; Mayoral et al., 2010; Figure 3F).

The *D. melanogaster* star1-RA and AlCR2 receptors are detected in the CNS and they are activated by allatostatin-C peptides, which are potent modulators of hormone synthesis (Aguilar et al., 2003; Hergarden et al., 2012). These peptides inhibit or stimulate the corpora allata to synthesize juvenile hormone, which is an important regulator of development and reproduction in insects and may indirectly influence feeding behavior (Audsley and Weaver, 2009; Nassel and Winther, 2010).

##### Galanin receptor subfamily

In *C. elegans* and *D. melanogaster* a sequence and function homolog of vertebrate GALR has been described (Figure 3F). The *C. elegans* GALR-like receptor, *npr-9* in common with the vertebrate homolog may be involved in food foraging and lipid storage (Bendena et al., 2008). The *npr-9* is expressed in specific neurons around the posterior pharyngeal bulb and *C. elegans* receptor mutants are characterized by impaired food-related roaming behavior and accumulate intestinal fat as a result of fat ingestion and reduced energy expenditure (Lang et al., 2007; Bendena et al., 2008). Peptides involved in the activation of *npr-9* have not been isolated, although *nlp-5* and *nlp-6*, are candidate allatostatin-like peptides that in insects activate the GAL-like receptor (Nathoo et al., 2001).

In arthropods, the Allatostatin type-A receptors are homologs of the vertebrate GALRs (Birgul et al., 1999). Two receptors have been described in *D. melanogaster*, DAR-1 (a.k.a. AlstR) and DAR-2 (Birgul et al., 1999; Lenz et al., 2000; Figure 3F). AlstR is expressed in *D. melanogaster* head and CNS while DAR-2 is expressed in the gut suggesting they may have divergent functions. The receptors are activated by FGLamide neuropeptides (Pratt et al., 1991; Woodhead et al., 1994) that in arthropods inhibit food intake (Audsley and Weaver, 2009). Genetic epistasis assays in *D. melanogaster* indicate that FGLamide neuron activation inhibits or limits starvation-induced changes in feeding behavior (Hergarden et al., 2012).

#### Novel subfamily members with an unknown role in feeding regulation

##### Neurokinin/neuropeptide FF/orexin-like receptor subfamily

In *C. elegans* four additional NKRs members may exist: *npr-14*, *npr-22* and the genes C49A9.7 and C50F7.1 (Keating et al., 2003). In *D. melanogaster* the SIFamide receptor and the gene CG10823 (Hewes and Taghert, 2001) also seem to be novel receptor members (Table 5, Figure 3B). In the phylogenetic tree, the *C. elegans* gene C49A9.7 clusters with *tkr-1* suggesting they may be duplicates and the nematode *npr-14* and C50F7.1 genes group with the fruit fly CG30340 and SIFamide receptor genes suggesting that they may have emerged from the same gene prior to the nematode-arthropod divergence. Functional studies of these receptors are scarce but those that exist indicate that the *C. elegans* MVRFamide neuropeptides but not tachykinin-like peptides activate the *npr-22* receptor (Mertens et al., 2006). The function of *D. melanogaster* CG30340 gene, which is present in low abundance in the digestive and nervous system and of SIFamide receptors are unknown (Jorgensen et al., 2006).

##### Neuropeptide Y-like receptor subfamily

In *C. elegans* at least eight putative novel NPYR gene members are predicted: *npr-3*, *npr-4*, *npr-6*, *npr-7*, *npr-8*, *npr-10*, *npr-12*, and *npr-13* and all remain to be validated and functionally characterized (Keating et al., 2003; Figure 3C). The receptors share between 30–40% amino acid sequence similarity with their human counterparts (Table 5) and are approximately 20% identical to the *C. elegans* homologs with a characterized function. The high sequence similarity and phylogenetic relationship between *npr-5* and *npr-13* (43%), *npr-4* and *npr-10* (50%) and *npr-11* and *npr-12* (44%) suggests that they may have arisen as a result of a recent duplication event in the nematode genome. These receptors are expressed in nervous tissue and intestine and their function is incompletely described and a specific role in feeding has not been demonstrated (Keating et al., 2003; Styer et al., 2008). In the *D. melanogaster* genome a putative novel insect NPY-like gene of unknown function (CG32547) may also exist (Hewes and Taghert, 2001) and seems to be expressed in the CNS (Figure 3C). The CG32547 gene shares less than 14% similarity with the human NPYR members (Table 5) and with the other insect family members, although this is probably due to its atypical size of 1008 amino acids, which makes family annotation ambiguous.

##### Ghrelin-obestatin/neuromedin U receptor subfamily

Two putative additional *C. elegans* nmur-like receptor genes the *npr-20* and *npr-21* were retrieved in the present study (Figure 3E). They share 30–36% amino acid sequence similarity with human homologs and are probably duplicates (Table 5). Expression of *npr-21* in *C. elegans* occurs in nerves of the head, tail, and ventral nerve cord and also in the posterior intestine suggesting that it may have a role in brain-gut function associated with feeding regulation. Similarly in *D. melanogaster* a putative member of this family was also retrieved, the gene CG34381 (Table 5) and it clusters with nematode *npr-20* and *npr-21* suggesting that it may have shared common ancestry (Hewes and Taghert, 2001). Expression of the CG34381 gene occurred in the fruit fly head but so far no functional studies have been reported.

##### Somatostatin receptor subfamily

In the *C. elegans* genome at least eight putative SST-like receptor genes are predicted: *npr-15*, *npr-16*, *npr-17*, *npr-18*, *npr-24*, *npr-32*, and the Y54E2A.1 (Vashlishan et al., 2008) and T02E9.1 genes (Keating et al., 2003; Figure 3F). No additional putative SST-like receptors were identified or have been reported for *D. melanogaster*. Characterization of the nematode putative SST-like receptors revealed the *C. elegans* members share between 27–35% amino acid sequence similarity with the human SSTRs and that the *npr-24* gene is the most closely related to the insect and human homologs suggesting that they may share a common ancestry (Table 5). Comparisons of the putative SSTR in *C. elegans* revealed they are highly divergent suggesting that after their emergence from an ancestral gene they underwent considerable change. Nematode *npr-17* is most similar to *npr-18* and to the T02E9.1 gene with which it shares 23% sequence identity and the three receptors tend to cluster with *npr-16* and *npr-32* suggesting they emerged in the nematode lineage.

The physiological role of the nematode SST-like receptors is poorly characterized but a role in metabolism and feeding behavior is probable. RNAi knockdown studies of *npr-16*, found to be expressed in head/tail neurons and the ventral nerve cord, increased fat deposition (Ashrafi et al., 2003). Ligand binding studies revealed that the peptide *nlp-3* activates the receptor *npr-17*, which seems to be involved in food aversion and has a role in serotonergic modulation via ASH sensory neurons to modulate nematode behavior in response to an external stimuli (Harris et al., 2010). Deletion of the T02E9.1 gene resulted in an uncoordinated phenotype and nematodes moved slowly and with an increase in circular movement, although feeding was apparently unaffected (Keating et al., 2003). The function of *npr-15*, *npr-18*, *npr-24*, *npr-32*, and Y54E2A.1 remain to be explored.

### Evolution of rhodopsin GPCR homologs in invertebrates

**Figure 4:**
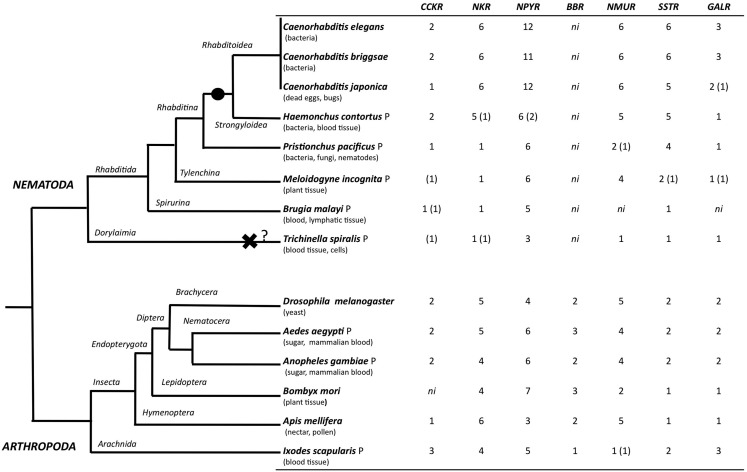
**Distribution of rhodopsin subfamily members in nematodes and arthropods**. The phylogenetic relationship of the species analyzed is represented on the right and their feeding habits are indicated. The black circle indicates a putative gene duplication event in the nematode radiation and the black cross potential gene deletion in the *T. spiralis* genome. Genes that were identified based upon sequence similarity but that were not considered for phylogenetic analysis are indicated within brackets “()”; ni- GPCR member not identified, and P represent parasitic nematode and arthropod. The evolutionary relationship within nematodes and arthropods was obtained from (Consortium, 2006; Sommer and Streit, 2011).

**Figure 5:**
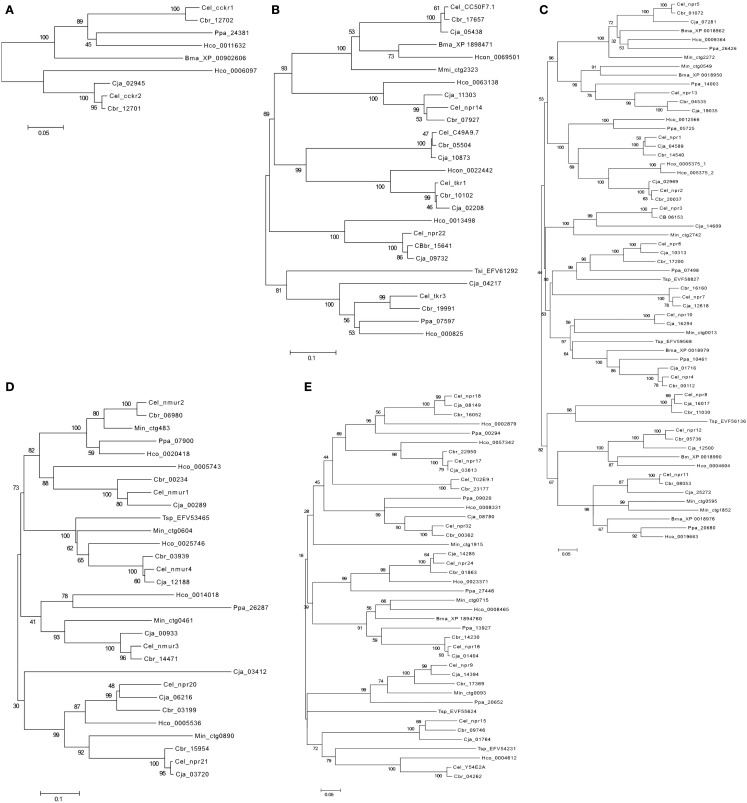
**Phylogenetic analysis of the nematode rhodopsin GPCRs**. **(A)** Gastrin-cholecystokinin receptors; **(B)** Neurokinin/neuropeptide FF/orexin receptors, **(C)** Neuropeptide Y receptors, **(D)** Ghrelin-Obstatin/neuromedin U receptors, and **(E)** Somatostatin and galanin receptors. The *C.elegans* (Cel) receptors are annotated in bold. *C. briggsae* (Cbr), *C. japonica* (Cja), *P. pacificus* (Ppa) *H. contortus* (Hco) *B. malayi* (Bma), *T. spiralis* (Tsp), and *M. incognita* (Min). Accession numbers of the sequences used are indicated. Trees were constructed using the sequence alignment displayed in Figure S1 Supplementary Material using the methodology described in Figure 3.

**Figure 6:**
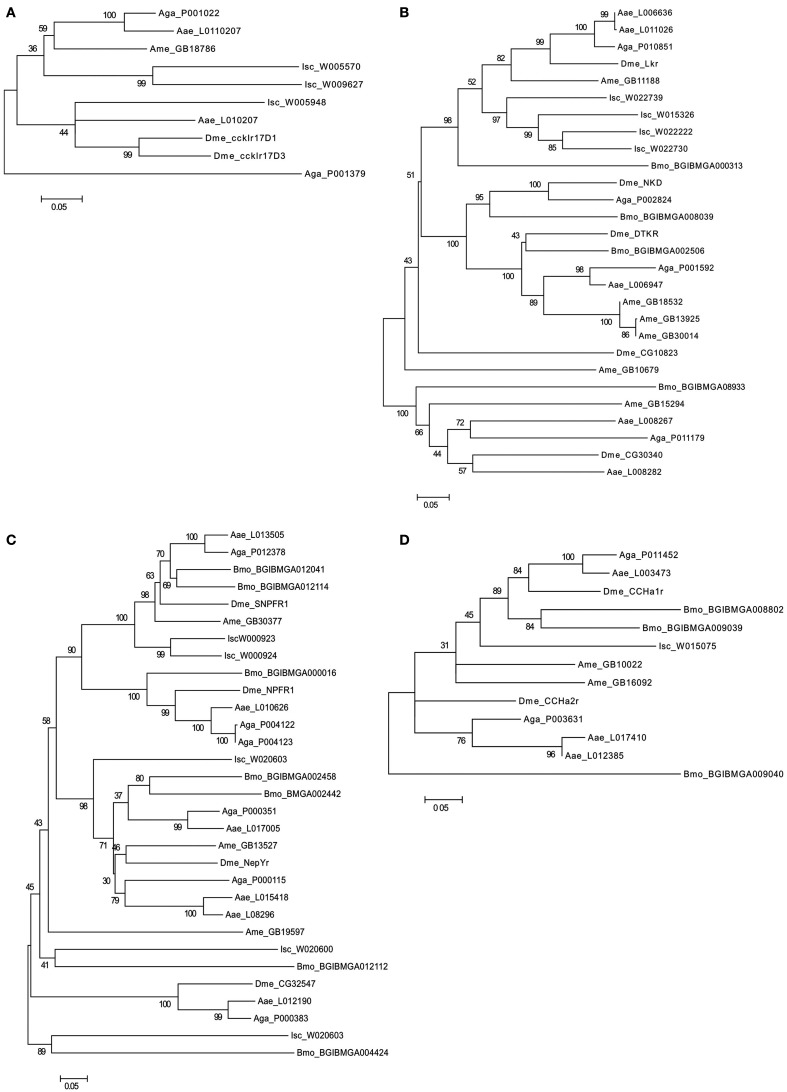

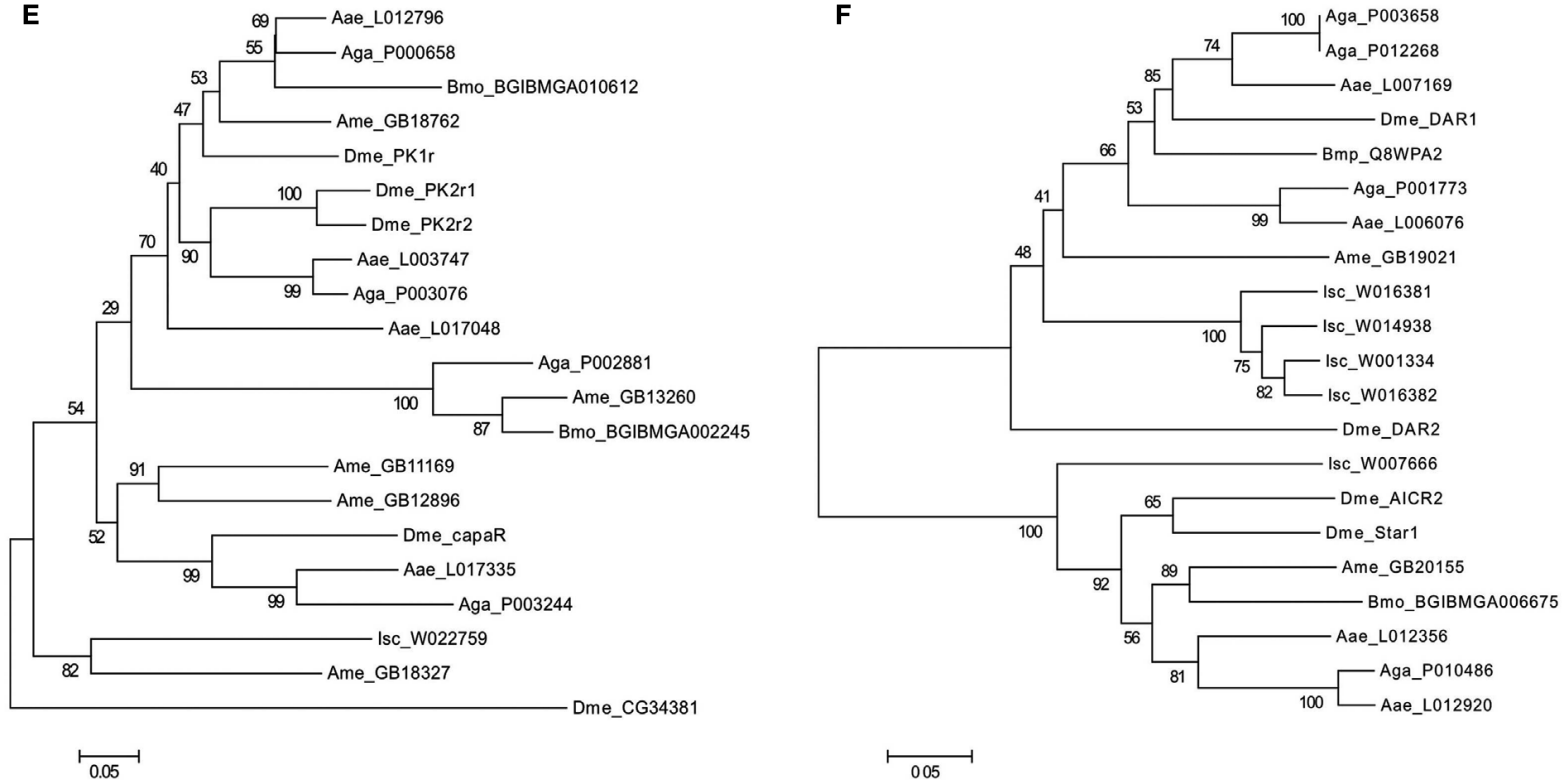
**Phylogenetic analysis of the arthropod rhodopsin GPCRs**. **(A)** Gastrin-cholecystokinin receptors; **(B)** Neurokinin/neuropeptide FF/Orexin receptors, **(C)** Neuropeptide Y receptors, **(D)** Bombesin receptors, **(E)** Ghrelin-Obstatin/Neuromedin U receptors, and **(F)** Somatostatin and galanin receptors. The *D. melanogaster* (Dme) receptors are annotated in bold. *A. gambiae* (Aga), *A. aegypti* (Aae), *A. mellifera* (Ame), *B. mori* (Bmo), and *I. scapularis* (Isc). Accession numbers of the sequences used are indicated. Trees were constructed using the sequence alignment displayed in Figure S2 in Supplementary Material using a similar approach to that described in Figure 3.

The evolution of the rhodopsin GPCRs in invertebrates was established (Figure 4) by identifying homologs in different nematode and arthropod lineages of the receptors present in *C. elegans* (Figure 5 and Table 6) and *D. melanogaster* (Figure 6 and Table 7). In general, the invertebrate GPCRs with a documented role in feeding or that are sequence homologs of mammalian seem to have evolved differently in nematodes and arthropods. A similar gene complement to that identified in *C. elegans* and *D. melanogaster* was identified in non-model nematodes and arthropods, respectively (Figure 4). Nematodes of the superfamily Rhabditoidea generally have more genes than other nematodes (Table 6). Gene duplicates in *C. elegans* and *C. brigssae* are more abundant than in arthropods (Lynch and Conery, 2000; Cutter et al., 2009) and a higher number of homologs of the human NPYRs and SSTRs occur in nematodes when compared to arthropods (Figures 5C and 6C). In arthropods, species-specific gene duplications exist rather than a conserved gene homolog complement suggesting that, despite their common ancestry, GPCRs have had distinct evolutionary trajectories in the different lineages (Table 7 and Figure 6).

**Table 6:** **Accession numbers of the *C. elegans* homologs in *C. briggsae*, *C. japonica*, *P. pacificus*, *H. contortus*, *B. malayi*, *T. spiralis*, and *M. incognita***.

Receptor subfamily	*C. elegans*	*C. briggsae*	*C. japonica*	*P. pacificus*	*H. contortus*	*B. malayi*	*T. spiralis*	*M. incognita*
Gastrin-cholecystokinin	*ckr-1* *ckr-2*	CBG12702 CBG12701	CJA02945	PPA24381	Supercontig0002945	XP_001902606	EFV58901*	MiV1ctg254*
					Supercontig0006097	XP_001895620*		
Neurokinin/neuropeptide FF/orexin	*tkr-1*	CBG10102	CJA02208	PPA07597	Supercontig0022442	XP_001898471	EFV61292	MiV1ctg2323
	*tkr-3*	CBG19991	CJA04217		Supercontig000825		EFV59206*	
	*npr-14*	CBG07927	CJA11303		Supercontig0069501			
	*npr-22*	CBG15641	CJA09732		Supercontig0013498			
	C49A9.7	CBG05504	CJA10873		Supercontig0000067*			
	C50F7.1	CBG17657	CJA05438		Supercontig0063138			
Neuropeptide Y	*npr-1*	CBG14540	CJA04589	PPA05725	Supercontig0012566	XP_001897991	EFV58827	MiV1ctg2742
	*npr-2*	CBG20037	CJA02969	PPA10461	SuperContig0005375	XP_001895072	EVF56136	MiV1ctg13
	*npr-3*	CBG06153	CJA14609	PPA26426	Supercontig0005375	XP_001896282	EVF59568	MiV1ctg2272
	*npr-4*	CBG00112	CJA01716	PPA07498	Supercontig0004842*	XP_001897675		MiV1ctg595
	*npr-5*	CBG01072	CJA07281	PPA20680	Supercontig0009364	XP_001899021		MiV1ctg1852
	*npr-6*	CBG17200	CJA10313	PPA14003	Supercontig0019663			MiV1ctg549
	*npr.7*	CBG16160	CJA12618		Supercontig0004604			
	*npr-8*	CBG11030	CJA16017		Supercontig0005938*			
	*npr-10*	CBG08053	CJA16294					
	*npr-11*	CBG05736	CJA25272					
	*npr-12*	CBG04535	CJA12500					
	*npr-13*		CJA19035					
Ghrelin-obstatin/neuromedin U	*nmur-1*	GBG00234	CJA00289	PPA17766*	Supercontig0005743	ni	EFV53465	MiV1ctg483
	*nmur-2*	CBG06980	CJA03412	PPA07900	Supercontig0020418			MiV1ctg461
	*nmur-3*	CBG14471	CJA00933	PPA26287	Supercontig0014018			MiV1ctg604
	*nmur-4*	CBG03939	CJA12188		Supercontig0025746			MiV1ctg890
	*npr-20*	CBG03199	CJA06216		Supercontig0005536			
	*npr-21*	CBG15954	CJA03720					
Somatostatin	*npr-24*	CBG01863	CJA14285	PPA27446	Supercontig0023371	XP_001894760.1	EFV55624	MiV1ctg715
	*npr-16*	CBG14230	CJA01404	PPA13927	Supercontig0008465			MiV1ctg1915
	*npr-17*	CBG22950	CJA03613	PPA00294	Supercontig0057342			MiV1ctg1587*
	*npr-18*	CBG16052	CJA08149	PPA09020	Supercontig0002879		
	*npr-32*	CBG00362	CJA08780		Supercontig0008331		
	T02E9.1	CBG23177					
Galanin	*npr-9*	CBG17363	CJA14394	PPA20652	Supercontig0004612	ni	EFV54231	MiV1ctg93
	*npr-15*	CBG09746	CJA01764					MiV1ctg1567*
	Y54E2A.1	CBG04262	CJA18843*		

*Indicates sequences not used in the phylogenetic analysis due to poor sequence or non-identification of TM domains. ni, indicates gene not identified.

**Table 7:** **Accession numbers of the *D. melanogaster* homologs in *A. gambiae*, *A. aegypti*, *A. mellifera*, *B. mori* and *I. scapularis***.

Receptor subfamily	*D. melanogaster*	*A. gambiae*	*A. aegypti*	*A. mellifera*	*B. mori*	*I. scapularis*
Gastrin-cholecystokinin	CCKLR-17D3	AGAP001022	AAEL010207	GB18786	ni	ISCW005570
	CCKLR-17D1	AGAP001379	AAEL017238			ISCW009627
						ISCW005948
Neurokinin/neuropeptide FF/orexin	NKD	AGAP002824	AAEL006947	GB13925	BGIBMGA008039	ISCW022739
	DTKR	AGAP001592	AAEL008267	GB30014	BGIBMGA002506	ISCW015326
	Lkr	AGAP011179	AAEL008282	GB18532	BGIBMGA008933	ISCW022222
	CG30340	AGAP010851	AAEL011026	GB15294	BGIBMGA000313	ISCW022730
	CG10823		AAEL006636	GB11188		
				GB10679		
Neuropeptide Y	NepYr	AGAP000351	AAEL017005	GB13527	BGIBMGA002442	ISCW020603
	NPFR1	AGAP000115	AAEL008296	GB19597	BGIBMGA002458	ISCW020600
	SNPFR1	AGAP000383	AAEL015418	GB30377	BGIBMGA000016	ISCW022779*
	CG32547	AGAP004123	AAEL012190		BGIBMGA012112	ISCW000923
		AGAP012378	AAEL010626		BGIBMGA012041	ISCW000924
		AGAP004122	AAEL007924		BGIBMGA012114	
			AAEL013505			
Bombesin	CCHa1r	AGAP003631	AAEL012385	GB10022	BGIBMGA008802	ISCW015075
	CCHa-2r	AGAP011452	AAEL017410	GB16092	BGIBMGA009039	
			AAEL003473		BGIBMGA009040	
Ghrelin-obstatin/neuromedin U	capaR	AGAP003244	AAEL017335	GB11169	BGIBMGA002245	ISCW012018*
	PK-1r	AGAP002881	AAEL012796	GB12896	BGIBMGA010612	ISCW022759
	PK2r2	AGAP000658	AAEL003747	GB18762		
	PK2r1	AGAP003076	AAEL017048	GB13260		
	CG34381			GB18327		
Somatostatin	Star1-RA	AGAP010486	AAEL012920	GB20155	BGIBMGA006675	ISCW007666
	AlCR2	AGAP012268	AAEL012356	
Galanin	DAR-1	AGAP003658	AAEL007169	GB19021	Q8WPA2	ISCW001334
	DAR-2	AGAP001773	AAEL006076			ISCW014938
						ISCW016381
						ISCW016382

*Indicates sequences not used in the phylogenetic analysis due to poor sequence or non-identification of TM domains. ni: indicates gene not identified.

A striking observation is the absence in nematodes of homologs of the arthropod bombesin receptors (BBR; Figure 4; Table 5). The reason for the loss of BBR in nematodes is unknown and their function and any link to feeding regulation remains to be established. In vertebrates, bombesin and its receptors are involved in smooth muscle contraction, exocrine, and endocrine secretion in the gut, pancreas, and pituitary and they also have a central role in food intake and energy homeostasis (Sano et al., 2004; Gonzalez et al., 2008). Three receptors have been isolated in humans and a similar number exist in arthropods and they share a common ancestry (Figure 6D).

A similar number of gastrin-CCK, NKR, NMUR, and GALR subfamily members were characterized in nematodes and arthropods (Figure 4). Two putative gastrin-CCK receptors were identified in invertebrates and in humans two gastrin-CCK receptors also exist suggesting that the evolution of the members of this family has been highly conserved. However, phylogenetic analysis suggests that the duplication, which delivered the two gene copies, was not common to all the species and occurred independently within each lineage. The two *ckr* that are present in nematodes resulted from a lineage specific duplication and homologs of the two *C. elegans* genes were identified in most nematode genomes analyzed (Figure 5A). In arthropods, a different situation exists and the two *D. melanogaster* genes are very similar and seem to have resulted from a species-specific duplication event (Figure 6A). Similarly in the blacklegged tick (*I. scapularis*) three putative gastrin-CCK receptors were also identified. In contrast, no putative homologs were identified in the plant feeding arthropod, the silkworm *B. mori*, even though they had a similar gene complement to other arthropods. It remains to be established if the absence of this receptor in *B. mori* is a consequence of its incomplete genome assembly (Xia et al., 2004) or represents an adaptation relative to feeding regulation.

Members of the NKR, NMUR, and GALR subfamilies have also evolved via lineage specific and species-specific duplication events. In nematodes, a similar number of NKR, NMUR, and GALR receptors exist in *H. contortus* and in the three representatives of the *Caernohabitis* genus analyzed (Figures 5B,D,E). In contrast, few genes of these families have been identified in other nematode taxa and a single NKR subfamily member was retrieved from *P. pacificus*, *M. incognita*, *B. malayi*, and *T. spiralis*. In arthropods, gene duplication of the *D. melanogaster* LKR receptor homologs was identified in the mosquito *A. aegypti* and also in *I. scapularies* in which four putative receptors exist (Figure 6B). In addition, in the honeybee (*A. mellifera*) three putative homologs of the fruit fly DTKR receptors were also identified. In contrast, no homologs of *D. melanogaster* NKD were detected in the honeybee and *A. aegypti* genomes. Within the NMUR family (Figure 6E), the *D. melanogaster* PK2Rs emerged as a consequence of a species-specific duplication event and two putative capaR were also identified in the honeybee, but only a single member was found in *I. scapulars*. In contrast, duplication of GALR occurred in the *I. scapulars* genome and four putative receptors were identified while other arthropods contained a single homolog of *D. melanogaster* DAR-1 and DAR-2 genes (Figure 6F).

The complete genome sequence of some of the species used in this study are not yet available, nonetheless gene representatives identified in the selected nematodes and arthropods provides a clear idea of the GPCR evolution in invertebrates. The majority of the *C. elegans* sequence homologs were identified in the target species and an increase in gene number seems to have occurred in Rhabditoidea and Strongyloidea (Abad et al., 2008; Dieterich et al., 2008; Mitreva et al., 2011). The exception was *B. malayi* in which representatives of NMUR and GALR were not identified possibly because of its incomplete genome assembly (Ghedin et al., 2007). The absence of the majority of the *C. elegans* receptor homologs in parasitic nematode genomes and the higher number of genes present in *H. contortus* and in other representatives of the *Caernohabitis* genus is curious. A general comparison of the gene content of *T. spiralis* with *C. elegans* revealed that the parasitic nematode genome contains fewer genes (15,808 compared to 20,060 and 19,507 in *C. elegans* and *C. briggsae*, respectively) and we hypothesize that gene absence is a consequence of the selective pressures provoked by the host on which they live and depend for survival (Mitreva et al., 2011; Sommer and Streit, 2011). The genome of *P. pacificus* is predicted to contain a higher gene number than *C. elegans* and suggests that a specific GPCR gene expansion occurred in the nematode lineage after their divergence (Dieterich et al., 2008; Sommer and Streit, 2011). Comparisons between *T. spiralis* and the other blood feeding parasitic nematode *H. contortus* revealed that the latter has a higher GPCR gene number than *T. spiralis*. One explanation may be related to their life cycles and while both nematodes need blood to survive *T. spiralis* is an obligate parasite, while *H. contortus* has a non-parasitic free-living stage. Intriguingly during the parasitic stage of *H. contortus* significant changes in the active transcriptome occurs when compared to the nematode free-living stage (Hoekstra et al., 2000) and it will be of interest to establish if this affects the diversity of rhodopsin GPCRs expressed.

In arthropods, GPCR gene evolution appears species dependent and specific gene duplications and deletions have occurred despite their common ancestry. The existence of specific gene duplicates in arthropods may indicate that a divergent regulatory system evolved in different species and the origin and maintenance of duplicates in the genome remain to be explored. Gene number in the two mosquito species analyzed are very similar and may reflect their identical life styles (Klowden, 1990). In the tick, which feeds exclusively on blood, a specific expansion of NKR and GALR gene families occurred. Further studies are required to determine the significance of the specific evolution of rhodopsin family GPCRs in arthropods and to consider how life style and feeding activity may have influenced receptor evolution.

## Final Considerations

In general, the physiological processes involving GPCRs are conserved and sequence and function homologs of vertebrate rhodopsin GPCRs are present in invertebrates indicating they emerged early in evolution. In Nematoda and Arthropoda the rhodopsin GPCRs have evolved differently. Gene expansion is observed in nematodes with a free-living stage and specific gene deletions seem to have affected parasitic nematode genomes. In arthropods species-specific gene duplications occurred. We hypothesize that the evolving feeding regime and life style of invertebrates was one of the pressure forcing GPCR evolution and that this may explain some of the specific gene family expansions and deletions. Comparative studies of GPCRs gained or lost in the nematodes and arthropods and their relationship to feeding regulation may provide insights into how GPCRs contributed and shaped adaptation to new ecological niche. Studies of other nematodes and arthropods coupled with experiments to assign function and potential conserved role in feeding will be needed to test this hypothesis.

## Conflict of Interest Statement

The authors declare that the research was conducted in the absence of any commercial or financial relationships that could be construed as a potential conflict of interest.

## Supplementary Material

The Supplementary Material for this article can be found online at http://www.frontiersin.org/Neuroendocrine_Science/10.3389/fendo.2012.00157/abstract

Supplementary Figure S1 **Sequence of the nematodes GPCR transmembrane (TM) domains from non-model nematodes within each receptor family were extracted by sequence homology using the roundworm *C. elegans* TM regions**. To facilitate visualization the TM1, 3, 5, and 7 were annotated in gray.

Supplementary Figure S2 **Sequence of the Arthropod GPCR transmembrane (TM) domains used in for phylogenetic analysis**. TM domains from non-model arthropods within each receptor family were extracted by sequence homology using the *D. melanogaster* TMs. To facilitate visualization the TM1, 3, 5 and 7 were annotated in gray.
