# Supplementary material for: Feeding and the Rhodopsin Family G-Protein Coupled Receptors in Nematodes and Arthropods
Source: Front Endocrinol (Lausanne). 2012 Dec 18;3:157. doi: 10.3389/fendo.2012.00157 (PMC3524798; doi:10.3389/fendo.2012.00157)
Supplement: Supplementary Figure S1 — Sequence of the nematodes GPCR transmembrane (TM) domains from non-model nematodes within each receptor family were extracted by sequence homology using the roundworm C. elegans TM regions. To facilitate visualization the TM1, 3, 5, and 7 were annotated in gray. [file 35103_Cardoso_Presentation1.PDF]

Supplementary Figure 1. Sequence of the nematodes GPCR transmembrane (TM) domains from non-model nematodes within each receptor family were extracted by sequence homology using the roundworm *C. elegans* TM regions. To facilitate visualization the TM1, 3, 5 and 7 were annotated in grey.

## A) Gastrin-cholecystokinin receptors

```
                    TM1                 TM2                TM3                TM4                 TM5                 TM6                 TM7
cckr1          ISSIFLLCLILSLLGNAIVILTILGLNLAFADLLRSIICIPSTQPVGVCASAYTLAVIAIERYYAIRALITISLVWCFSFSANLTSLFQLYLTFTLLFVPLALMVGLYGNVIVTRMLITLVIVFAFCWVPSYLYWLLNSSLTIMTYISSLANPITYCFMNKSFR
cckr2          IAVTFFMIFLLSVVGNSVVLIVIIKMNLAASDMMLSVVCMPPTQPVVVTASAYTLAVIAFERYFAIHAYAMITLVWVIAIAANILMLFQVYMTVVLLVIPLVVMAGLYGNVIVIKMLIVVVIIFFCCWTPSYIWWLLNTFITLLTYISSCTNPITYCFLNKKFR
CBG12702       ISAVFILCLILSLLGNAIVILTILGLNLAFADLLRSIICIPSTQPVGVCASAYTLAVIAIERYYAIRALITISLVWCFSFSANLTSLFQLYLTFTLLFVPLALMVGLYGNVIVTRMLITLVIVFAFCWVPSYMYWLLNSSLTILTYVSSLANPITYCFMNKSFR
CBG12701       IAVTFFTIFLLSVVGNSVVLIVIIKMNLAASDMMLSVVCMPPTQPVVVTASAYTLAVIAFERYFAIHAYAMITLVWVIAIAANILMLFQVYMTVVLLVIPLVVMTGLYGNVIVIKMLIVVVIIFFCCWTPSYIWWLLNTFITLLTYISSCTNPITYCFLNKKFR
CJA02945       IAVTFFIIFLLSVIGNSLVLIVIIKMNLAASDMMLSVVCMPPTQPVVVTASAYTLAVIAFERYFAIHAYAMITLVWTIAILANFLMLFQVYMTVVLLVIPLVVMTGLYGNVIVIKMLIVVVIIFFCCWTPSYIWWLLNTFITLLTYISSCTNPITYCFLNKKFR
XP 001902606   --------------------------------MFRSIICIPPTQPVTVCASAYTLALIALERYYAIRLLALISFVWLTAFGSNIGALVQIHVTLLLLFIPLTIMLLLYCKVIVIRMLIIIVIAFVICWSPSFIWWLTNAYITLLTHISACANPITYCFMNERFR
XP 001895620   -----------------------------------------------------------------------------------------------------------------VIKMLIVIVIIFFCCWTPNYMWWLLNTAITVLCYISSCANPITYCFLNKKFR
SC0011632      ---------------------------- FLLFVSSVVCMPLTQIVGVCASAYTLAVIAVERYYAIRALITISMVWLFSFICNIGSLFQLYVTLVLLFIPLCAMVSLYGHVI---------------------------------------------------
SC0006097      VVFTFITIFLLSVIGVIVVIMQLFQLNLAITDLMLSVVCMPPTQTILRYMPPIAFTNMANQVRDLRHAYAMIMLVWVVALAANVLMLFFVYMTIVLLAVPLVIMTVLYGSVITYRMLVVVVIIFFCCWTPSYIWWLLNTFITLLTYISSCTNPITYCFLNKKFR
MiV1ctg254     -----------------------------------------------------------------------------------------------------------------LIRMLIVIVVLFFFCWTPSYIWWIFNTIITVLTYLSSCTNPITYCFLNNKFR
EFV58901       LMVVFTIIFCLSVIGNSLVIVTIVQLNMAISDLLLTLICMPPTMSVSVAANANTLVAIALERYYALNVVRMMIIVWTASFTCSIPQAIFIFLSLVLFIIPLIIMTMLYSLVIVIKMLAAIVAEFFICWMPFYTFYLVYVTFLIMAYLSTCTNPITYCFMNSKFR
PPA24381       LSVVYLLLFLFSVLSNVFVIITILGLLLHHTTHSRSLVCIPSTQPVGVCASAYTLAVIAVERYYAIRAVILLIGVWAFSFITNVASLLQLYITLVLLFIPLSLMIYLYGNVIVTRMLITIVVLFALCWTPNYIWWLVNTALTLLTYVSSCTNPLTYCFMNKSFR
```

## B) Neurokinin/ neuropeptide FF/ orexin receptors

```
tkr1           FVAIAFVLLMATAIIGNSVVMWIIYFNMAFADLLIALFNVGTSGIAPTTVSVCSMMALSWDRCQAVRSVIAILIIWVVSTVTALPFAIEKVLFGIQYALPIIILGSTFTRIAAVKMLFLMVVAFVVCWLPYHIYHAFYLLIYWIAMSSCAYNPIIYCFANERFR
tkr3           IWCMLYAIIAFMAVVGNLLVLYITLLNLGFADLFTGIFAIPFKETVALTVSVFTLVTSAVHEFRTMSAKRCVLLIWIMAVLVSLPHGLNVYLVIIQYFVPMIILDTAYTMIALMRTLIIVVACFSLCWFPLETYLLLFFFSHWLAMSNSCLNPIIYGLYNTKYN
npr14          WFVYILASMMVIGVIGNTLVVVVVAMNLAIADLLILLFCLPLTNNTSVYVSIMSLVFITCERWRAIRTRSVIGGIWFIAMFLSSPEPVQLLQTIFSFVLPLLVISILCLHMVAVRMLCAVVFLFSMSNLPVHLYNIAKLLPRVFSYSSSCLNPILYSFLSGRTI
npr22          LCILFYSILCVCCVYGNVLVILVIVLNLAVADLLISVFCIPFSQAMAVFLSAWTLVVISFDRWMAIRALYLVAATWIFSILMALPLLFSSMVLILQYVVPQAVLIITYTHIGLVPMVILISALFALCWLPLLILINVWWFAHGLAMSHSMVNPIIYFIRNARFR
C49A9.7        FYGILFTLTIFLALMGNFTVMWIILFNLAVADASISVFNTGFSGITPICASVFTMIVMSIERYYAISTVTIIIMIWFMAFLFGVPAFLNNGLITVQYILPLCILSAAYYRVGASIMLAVVVFIFMIVWFPYNAYYLTYINIYWLGMSSTVFNPVIYYFMNKRFR
C50F7.1        LYTVAYGAVFFTGVLGNTFVVLAVWLSLALADLFILWICLPTTNASTSFASVYTLVAVTADRYLAIYTKYVIFAVWLVAAIFGIPNWYLFVNLLLAFIVPFGLISGLYTRIFVATMMLTVIIVFACCWLPLYCIFTYRPIFQWMSLLSSSLNPIIYIAYSHKYR
CBG10102       FVAIAFVLLMATAIIGNSVVMWIIYFNMAFADLLIALFNVGTSGIAPTTVSVCSMMALSWDRCQAVRSVIAILVIWVVSTVTALPFAIEKVLFGIQYALPIIILGSTFTRIAAVKMLFLMVVAFVVCWLPYHIYHAFYLLIYWIAMSSCAYNPIIYCFANERFR
CBG19991       IWCFLYILIAFMAVVGNLLVLYITCLNLGFADLFVGIFAIPFKETVALTVSVFTLVVSAVHEFRTVSAKICILLIWVLSVVVSLPHGLNIYLVVIQYFIPMLILDTAYAMIALMRTLIIVVACFTFCWFPLETYLLLFFFSHWLAMSNSCLNPIIYGLYNTKYN
CBG07927       WFVFILGTMMVIGVVGNALVVVVVSMNLAIADLLILLFCLPLTNNTSVYVSIMSLVFITCERWRAIRTRSVIAGIWLLAMFLSSPEPVQLVQTIFSFVLPLLVISVLCLHMVAVRMLCAVVMLFAISNLPVHLYNIAKLLPRVFSYSSSCLNPILYSFLSG---
CBG15641       LCILFYSILCVLCVYGNVLVILVIVLNLAVADLLISVFCIPFSQAMAVFLSAWTLVVISFDRWMAIRALYLVAATWIFSILMALPLLFSTMVVILQYIVPQAVLIITYTHIGLVPMVILISALFALCWLPLLILINVWWFAHGLAMLHSIVNPVIYFIRNARFR
CBG05504       FYGILFTMTIFLALMGNFTVMWIILFNLAVADASISVFNTGFSGITPICASVFTMIVMSIERYYAISTVTIIIMIWFLAFLFGVPAFLNNGLITFQYILPLCILSAAYYRVGASIMLAVVVFIFMIVWFPYNAYYLTYINIYWLGMSSTVFNPVIYYFMNKRFR
CBG17657       LYTVAYGAVFFTGVLGNTFVVLAVWLSLALADLFILWICLPTTNASTSFASVYTLVAVTADRYLAIYTKYVIFTVWFVAAIFGIPNWYLFVNLLLAFIVPFGLISGLYTKIFVATMMLTVIIVFACCWLPLYCIFTYRPIFQWMSLLSSSLNPIIYIAYSHKYR
CJA02208       FVAISFVLLMATAIIGNSVVMWIIYFNMAFADLLIALFNVGTSGIAPTTVSVCSMMALSWDRCQAVRSVTAILVIWVLSTVTALPFAI-----------------------------------------------------------------------------
CJA04217       -------------------------TTLPDRAIRGNRRPNSFRSKVCEKMRFVDMDNVGDCVVATREFDYCAELLMLQ----------NVYLVIIQYFVPMIILDTAYTMIALMRTLIIVVAAFSLCWFPLETYLLLFFFSHWLAMSNSCLNPIIYGLYNTKYN
CJA11303       WFVIILAGMMVIGVIGNTLVVVVVAMNLAIADLLILLFCLPLTNNTSVYVSIMSLVFITCERWRAIRTRSVIAGIWLLAMFLSSPEPVQLVQTISSYVLPLGVISVLCSHMVAVRMLCAVVFLFALSNLPVHLYNIA---------------------------
CJA09732       LCILFYSVLCVCCVYGNVLVILVVVLNLAVADLLISVFCIPFSQAMAVFLSAWTLVVISFDRWMAIRALYLVAATWIFSILMALPLLFSSMVVILQYIVPQAVLIITYTHIGLVPMVILISALFALCWLPLLILINVWWFAHGLAMLHSIVNPVIYFIRNARFR
CJA10873       FYGILFTLTIFLALMGNFTVMWIILFNLAVADASISVFNTGFSGITPICASVFTMIVMSIERYYAISTVTIIIMIWLLAFLFGVPAFLINGLITVQYILPLCILSAAYYRVGASIMLAVVVFIFMIVWFPYNAYYLTYINIYWLGMSSTVFNPVIYYFMNKRFR
CJA05438       LYTVAYGAVFFTGVLGNTFVVLAVWLSLALADLFILWVCLPTTNASTSFASVYTLVAVTADRYLAIYTKYVIFTIWFVAAIFGIPNWYLCVNLLLAFIIPFGLISGLYTKIFVATMMLTVIIVFACCWLPLYCIFTYRPIFQWMSLLSSSLNPIIYIAYSHKYR
XP 1898471     -------------------------------------------NSTTSCASIFTLVAVTADRYLAIWTLYVIAGIWMLSGILAAPTLAIIVNLLLAFTIPFILISALYTRIFVAQMMFTVIIVFALCWLPLHGIYSYRPIFQWLSLLSSSLNPLIYIVYSQKYR
SC0022442      -------------------------FNMAFADLLIAVFNVGTSGIAPTTVSVFSMMALSWDRRCQARSLITIAIIWAISTVTALPFAFDHILFLIQYALPLFVLSLTFARIA---------------------------------------------------
SC000825       -------------------------INLAFADLLTGIFAIPFKETVSLSVSVFTLTASAVHEFRTTSAREIVVFLMALIHTS------NVYLTIIHYFVPMIILDTAYTMIALMNMLMIVVACFSLCWFPLETYLLLFFCSHWLAMSNSCLNPIIYGLFNLLQN
SC0063138      -------------------------------------------QNTSVYVSILSLMFITFERWRAIATRYIIAG-------------QIVLTLCAYLSPLLFITVLCLHMSAVRMLIAVVCMFALSYLPVHLHNIAKLLPRVFSYSSSCLNPVLYNFM-----
SC0013498      LCICFYNILCVCSVYGNFLVILVILFNLAIADLLIAVFCIPFSQGIAVFLSSWTLVVISFDRWMAIRAVYLVLATWAFRLAPSPFPLFSYLILVLQYVVPLTVLIITYTFIGLIPMVILISAMFAFCWLPLLLLINVWLPLLRFLTLLPQLFPHDWHVLH----
SC0000067      RWAGKTEGVYENPWGWTNFIVCFKMAKNARKLEFVGLGLSLVSLMIAILMVVIIRLVLYIDLIFTGINTMIICRLFFFLRCLHLLRVLGAEQLLSNYLKGEFVNLFFICNVILIPLLGIPNIMQTIPFAPTRDNITVITIIYCFTNKEVSVIQVNTVLKAFYDR
SC0069501      --------------NFFIILNWLIWYRNRACCLFDIRWDPSKSNATTSCASIYTLVAVTADRYLAIYTLYVIAGIWVVSGSLATPSLY------------------------VATMMLTVIVVFALCWLPLYGQFFF--------------------------
MiV1ctg2323    VFAFFYAAVFFGGFVGNMFVVVAIAVALKFADLLILLICLPSTNSTTSCASIFTLVGVTGERYL------------------------------------------------------VIVVFVLCWTPLYGLYCYRPIFQALFLFQWLSLLSSSLNPLIYIC
EFV61292       IWFILYAVIAFLTVAGNALVIYVFFVNLAVADLMTGLLAIPFKQYRCSRSACLQSTDSGRWSPAVTSVYLTGLTIECSS-------------------------------LGQTTQGPCACLCTARKPGGKLTTC--------------------------
EFV59206       -MNPLRKRMSRHKTIATIVVIWIAA-------MFLHSYTKKFFDGDAKTSAQFKIYNYFLLFITYIARVLWGGKTIGEHRQESQ---------------------------VVKMLTVVGSLFMVCWLPYHIYFTFYMSMYWLAMSSTVVNPIIYCWMNSRFR
PPA07597       LLCLLYAIIAFLGFAGNMLVVIVTLINLAIADLLTALLAIPFKETVSLSVSVFTLTASAIHEFRTVNARILVILIWLFALLVSVPHGVNIYLVMIQFFIPMIILDTAYTMIALMKMLMIVVLCFTLCWAPLETYLALFFFSHWLAMSNSCLNPIIYWFFNAKYK
```

## C) Neuropeptide Y receptors

```
npr1           PFLTVYLFLFFLGLFGNVTLIYVTCLNLAASDCMMCILSLPITQGISIFVCTFSLGAIALDRYILVGAFLTTVLLWILSFVVTLPYAFTMIVMLAQFVVPFAVMAFCYANIVTTSILVTMVVWFGITWLPHNVISLINLFTHSIAMSNNVLNPVLYAWLNPSFR
npr2           TFSLLYLHIFLLGILGNSAVLYLTMLNLCASNVLMCLTSLPITQGASIFVSTFSLSAIALDRYNLVSAMMIALLIWVISVVVCMPYGWTFLVLITQFLFPFATMAFCYYNIFTTTILSCMVLLFAFTWLPHNVVTLMSMTAHLISMLTNVTNPFLYAWLNPMFK
npr3           IFSLLYLLVWVGAIVGNTLVLYVLTGCLAGSDLLMCLFSLPITQGGTIFVSSFTLTVIALDRCVLIRAVFIVFCIWLLGYSLALPVGIGLSVLVLQFGIPALISSICYWMISANRMMIVMVVGFVLAWMPFNAVNLYFALCHVCAMCSAVLNPIIYSWFNPQFR
npr4           VFAFLYLIIIAAGIIGNSCVILAITLSLSCSDIVVCCTSATITAGISLCFSTFTLTAISIDRYILIQAVGVIAIICAFAATITSPIMFGAALMFLQLVIPLTIIIISYTAISTNRMLIGMVVAFACSWIWSVTFNILGIATHCIAMTSTVWNPLLYAVLNLQLR
npr5           IFTFLYGFLFVLGIFGNGGVLWAVALNLIFTDLILVFTAIPVTNSCSVFVTSWSLTAISLDKFLHIQALAITFLIWIVSTLINLPYLMGTTVMLLQFVVPMAVITYCYFKILVNYILIAMVVTFIGCWLPLTLLNLVAINAHVIAMSLVVWNPLLFFWLTRKQK
npr6           FFISMYCAVFLVASSGNFLVVYVVMTNLAVSDIMVNFTSLWLTQGTSIFISTWTLTAIAIDRYIVISCLSFIVLIWLCSLLLVTPYAIGIVVMILQFILPFVLIAISYIKIWLLRMLIVMVVIFAICWFPFNLLNCLFLSVHLMSMTATAWNPILYAFMNETFR
npr7           LLAVGYGLVGALSLVGNLAVLLIVISSVSAADLVITSFSLWATQGLSLMWVPLTLAAVALDRYSLVTCLLIIAGIWMGGFAVLSPMIRGLSVLVIRSAIPLVLISLCHWRIALQTLLLAMVVIFAVSSLPLDLSNVLFFFCHWTAMAGTLLNPLVYAYYNENFR
npr8           IYTTVYIIISVAAVIGNGLVIMAVVLNLALSNLILAITNIPFLPGSNIYCSTLTISVMAIDRYYSVHAVLVSLAIWIVSFILSLPLLLQLLMSILQVAFLYIVPLFVLSIFNTTSLLIAMAGSYAALWFPFTLITFLDQTCKMVSMLSICVNPFLYGFLNTNFR
npr10          ILATIYTIIVVVGVTGNLLVVMSVMVSLSVSDIFVAIVSGSVTQGTALSFSTLTLTAIAIDRYILIQALKMISFNSAISVGLSVPLFMGTVVFIIQFVFPLITITFCYASISTNRMLIIMTVTFALSWLPSVGFNFLGIIFHCISMTSVIVNPFLYGYCNEHFR
npr11          FITFAYMLIILFGAIGNFLTIIVVILNLALSDFFVCIVTAPTTQGFNIFLSTFSIASIAVDRYVLILSFCFFIMIWVISLILAVPLLQTLAVLITQYAFPLFSLVFAYSRIATHLLLVCVVAVFAVAWLPLNVFHIFFSICHCLAMCSACLNPLIYAFFNHNFR
npr12          PIVPMYGLVCSFGALANFIVLLAFVVNLAFSDLILCVVTAPVTQAVNTFVSSLTLAFIAMDRVLLTAPLLCYGVVWIISIIVALPYALTFVVLGIQYILPLAALAYAYFQIGALLLLFLLVLTYAVCWAPMNIYHVLYIFCHLVGISSTCVNPIVYALVNESFR
npr13          GYSVLYFLILIIGLVGNGLLITSILINLAVSDLLLCITAVPITQAFSVLISSWSLCYIAIDRYRSIHARWLLMFTWVVAFLASYPLYYTTSLLIIQLIIPAIIMSFCYLMILVMYVLILMVIVFMACWFPLSAVNLFLLNVHVIAMTSIVWNPVLYFWMSKRHR
CBG14540       PFLCVYIFLFFLGLFGNVTLIYVTCLNLAASDIMMCILSLPITQGISIFVCTFSLGAIALDRYILVGAFLTTILLWILSFVVTLPYAFTMIVMLAQFVLPFAVMAFCYSNIFTTSILVTMVVWFGITWLPHNVVSLINLFTHSIAMSNIVVNPVLYAWLNPSFR
```

| | |
|---|---|
| CBG20037 | SFSIMYLHIFLLGILGNSAVLYLTMLNLCASNVLMCLTSLPITQGASIFVSTFSLSAIALDRYNLVSAMMIALLIWVISVVVCMPYGWTFLVLITQFLFPFATMAFCYYNIF-------------------------------MLTNVTNPFLYAWLNPMFK |
| CBG06153 | IFSVLYLIVWVAAIVGNTLVLYVLTGCLAGSDLLMCLFSLPITQGGTIFVSSFTLTVIALDRCVLIRAVFIVFCIWLLGYCLALPVGIGLSVLVLQFGIPAFISSICYWMISANRMMIVMVVGFVLAWMPFNAVNLYFALCHVCAMCSAVLNPIIYSWFNPQFR |
| CBG00112 | IFAFLYLIIIAAGIIGNTCVILAITLSLSCSDIVVCCTSATITAGISLCYSTFTLTAISIDRYILIQALGVIALICAFAATITSPIMFGAALMFLQLVIPLTIIIISYTAISTNRMLIGMVVAFACSWIWSVTFNILGIATHCIAMTSTVWNPLLYAVLNLQLR |
| CBG01072 | IFTFLYGFLFVLGIFGNGGVLWAVALNLIFTDLILVFTAIPVTNSCSVFVTSWSLTAISLDKFLHIQALGITSLIWIVSTLINLPYLMGTTVMLLQFVVPMAVITYCYFKILVNYILIAMVVTFIGCWLPLTLLNLVAIVAHVIAMSLVVWNPLLFFWLTRKQK |
| CBG17200 | AFISAYFAIFCVAIFGNFLVIYVVMTNLAVSDLMVNFTSLWLTQGTSIFISTWTLTAIAIDRYIVISCLAIIVFFIWGASLLMVAPYGIGLVVMTLQFIVPFVLIAISYTKIWLLRMLICMVVIFAICWFPFNLLNCLFTPVHLISMTATAWNPILYALKNDTFR |
| CBG16160 | LLAFAYGSVGALSLVGNLAVLLIVVSSVSAADLVITSFSLWATQGLSLMWVPLTLAAVALDRYCLVTCLLIIAGIWLGGFAVLSPMIRGLSVLVIRSAIPLILISICHWRIALQTLLLAMVVIFAVSSLPLDLSNVLFFFCHWLAMAGTLLNPLVYAYYNENFR |
| CBG11030 | LYTTVYIIISVTAVVGNGLVILAVILNLALSNLILAITNIPFLPGSNIYCSTLTISVMAIDRYYSVHAILVSVAIWIVSFILSLPLLLQLLMSILQVVFLYIVPLFVLSIFNTTSLLIAMAGSYAALWFPFTLITFLDQTCKMVSMLSICVNPILYGYLNTNFR |
| CBG08053 | FITFAYMLIILFGAMGNFLTIVVILNLALSDFFVCIVTAPTTQGFNIFLSTFSIASIAVDRYVLILSFCFFIMIWVISLILAVPLLQTLAVLVTQYAFPLFSLVFAYSRIATHLLLVCVVAVFAVAWLPLNVFHIFFSICHCLAMCSACLNPLIYAFFNHNFR |
| CBG05736 | PIVPMYGLVCSFGALANFIVLLAFAVNLAFSDLILCVVTAPVTQAVNTFVSSLTLAFIAMDRVLLTAPLLCYVFVWIISIMVALPYALTFVVLAIQYILPLVALAFAYFQIGALLLLFLLVLTYAVCWAPMNIYHVLYIFCHLVGISSTCVNPIVYALVNESFR |
| CBG04535 | AYSSLYTFIFVTGVIGNGLLISSILINLAVSDLLLCITAVPITQAFSVLISSWSLCYIAIDRYRSIHARWLLIFTWVVAFLASYPLYYTTSLLIIQLIIPAIIMSFCYWKIL-------------------------FSNCPHVGV----------------- |
| CJA04589 | PFLCIYLFLFFLGLFGNLTLIYVTCLNLAASDSMMCILSLPITQGISIFVCTFSLGAIALDRYILVGAFLTTVLLWILSFVVTLPYAFTLIVMLAQFVVPFAVMAFCYANIFTTSILVAMVVWFGITWLPHNVISLINLFTHWIAMSNNVVNPVLYAWLNPTFR |
| CJA02969 | -------------------------LNLCLSNVLMCLTSLPITQGASIFVSTFSLSAIALDRYNLVSAMMIALLIWVISVVVCMPYGWAFLVLITQFLFPFATMAFCYYNIF-------------------------------------------------- |
| CJA14609 | VFSLLYLFWVAAIVGNTLVLYVLTGCLAASDLLMCLFSLPITQGGTIFVSSFTLTAIALNQCGLIPARCLYRLLHMASRLLISPPCRGLLVLVLQFGIPAVISSICYWMISANRITAKSEDYLEESQAGFRRGRGCVFLDFTCAFDNVNWTKISQVLNNLQIG |
| CJA01716 | IFAFLYMIIIAAGIIGNTCVILAITLSLSCSDIVVCCTSATITAGISLCYSTFTLTAISIDRYILIQAYCVIGLICAFAATITSPIMMGAALMFLQLVIPLTIIIISYTAISTNRMLIGMVVAFACSWVWSVMFNILGIATHCIAMTSTVWNPLLYAVLNLQLR |
| CJA07281 | IFTFLYGSLFVLGIFGNGGVLWAVALNLIFTDLILVFTAIPVTSQIFAYQRSYKATCIGNSPFFPTQASLITFLIWIVSTLINLPYLMGTTVMLLQFVVPMAVITYCYFKILVNYILIAMVVTFIGCWLPLTLLNLVAMIAHVIAMSLVVWNPLLFFWLTRKQK |
| CJA10313 | FFIAMYCVIFAVASSGNVLVVYVVMTNLAISDLMVNFTSLWLTQGTSIFISTWTLTAIAIDRYIVITCLLFIVTIWLCSLLLVTPYAIGLVVMFLQFILPFVLIAVSYTKVWLLRMLIVMVVIFAICWFPFNLLNCLFLSVHLMSMTATAWNPILYAFMNETFR |
| CJA12618 | MLAVAYGLVGALSLVGNLAVLLIVVSSVSAADLVITSFSLWATQGLSLMWVPLTLAAVALDRYSLVTCLLIIAGIWIGGGFAALSPMIRGLSVLVIRSAIPLILISLCHWRIALQTLLLAMVVIFAVSSLPLDLSNVLFFFSHWTAMAGTLLNPLVYAYYNENFR |
| CJA16017 | LYTTVYIIISVAAVIGNGLVILAVVLNLALSNLILAITNIPFLPGSNIYCSTLTISVMAIDRYYSVHAVLLSVFIWILSFILSLPLLLQLLMSILQVVFLYIVPLFVLSIFNTTSLLIAMAGSYAALWFPFTLITFLDQTCKMVSMLSICVNPFLYGFLNTNFR |
| CJA16294 | ILATIYTIIVVVGCSGNLLVVMSVVVSLSVSDIFVAIVSGSVTQGTALSFSTLTLTAIAIDRYILIQALKMIIFNSGISIGLSAPLFMGTVVFIIQFVFPLITITFCYASISTNRMLIIMTVTFALSWLPAVGFNFLGIIFHCISMTSTIVNPFLYGYCNEHFR |
| CJA25272 | FITFAYMMIVLFGAIGNFLTITVVVLNLALSDFFVCIVTAPTTQGFNIFLSTFSIASIAVDRYVLILSFCFFVMIWITSLVLAVPLLQFFEKLYIVFSIDLRHEYAKLRPLI-------------------------------------------------- |
| CJA12500 | PIVPMYGLVCSFGAIANFIVLLAFIVNLAFSDLILCVVTAPVTQAVNTFVSSLTLAFIALDRVLLTAPLLCYLVVWIISIMVALPYALTFVVLAIQYVLPLAALAFAYFQIGALLLLFLLVLTYAVCWAPMNVYHVLKFLNFKFQVHILPPCPTISTLLRHDHR |
| CJA19035 | AYSSLYTFIFVTGVIGNGLLISSILINLAVSDLLLCITAVPITQAFSVLISSWSLCYIAIDRYRSIHARWLLFVTWIGAFLASYPLYYTTSLLIIQLIVPAIIMSFCYWKIL-------------------------FAEKSSVPPEIEQKNEAKVRKSKF--- |
| XP 001897991 | IFVFFYAAIILIGIVGNLCVILAISFSLSCSDLVVCFTSATITAGASLCFSTFTLSAISVDRFLLIQALIVIMVICMLSTSLSAPVIFGSIMLSVQFIVPLVIITFCYTAISTNRMLIGMVVAFSASWFFSVLFNVLGIATHCIAMSSTVWNPLLYAALNLQLR |
| XP 001895072 | --------------------------------------------------------------------HAYSLMAISWLTSATVSSPLFVGSMLLICQYLIPVTIMTFCYWKILVMYVLILMVVVFMASWLPLTIVNIFLLNVHAIAMTSIVWNPLLYFWMSK--- |
| XP 001896282 | --------------------------------------------------------------------QAGLVTAFIWVVSTMINIPYLMGSSVVLLQFVIPLTITYCYARILVNYILIGMVIAFIGCWLPLTVVNMLPLLAHVIAMSTVIWNPLLFFWLTKR-K |
| XP 001897675 | ---------------------------------MFLSFRFRNTLGFGIFLSTFSIAAIALDRCVVILALLFFLIWVISITLALPLLITLAVLLTQYALPLISIVFAYSTIATHLLLICVVIVFAVAWLPLNVFHVLFAICHLIAMGSACLNPVSYAFFNQNFR |
| XP 001899021 | -------------------------VRPAMSDFLLCVVTAPVTQAVNMFVSCMTLVLIAMDRVLLTAPVMCYCTVWLASIVIAAPYFFTSLMLAIQYLLPLAALAFSYSQIGALLLLSLLVLIYATAWFPMNAYNVLYLFCHLVGMTSACINPIVYALINDSFR |
| SC0012566 | LFAVLYVIY----------------VNLAVSDVILCVLSIPLTQAIGLFIGTFSLCAIAIDRFRLVNAIRITILLWIISIIVTLPYVYTLFVLAIQYVIPFTIMTICYQAVSVTLILVSMVVIFGITSLPHNIVSTLITHILNLAMLSCVANPMLYAFLNPEFR |
| SC0005375 | -------------------------LNLAASDVLMCLTSLPIT----------------------GASVVALLLWIISALVCMPYGWALMVLVMQFILPFATMAVCYYTIF-------------------------ISDCSISMTTNVANPILYAWLNPSFK |
| SC0005375 2 | IFAVLYVHIFVLGIVGNVAVLVYFHLNLAASDVLMCLTSLPITQQASVFVSTFSLSAIALDRYNLVGASVVALLLWIISALVCMPYGWALMVLVMQFILPFATMAVCYYTIFTWLPHNVYTLIIEYDEAFFHNGESDLFVLISISMTTNVANPILYAWLNPSFK |
| SC0004842 | IFAILYSVIILCGFIGNVCVIVAITLSLSFSDIAVCSISATITAGISLCFSTFTLTAISIDRYLLIHAV------------------------------------------------------------------------------------------- |
| SC0009364 | AFTLVYGLLFILGLVGNGGVVFAMALNLILTDLLLVLTAVPVTPSAMCHIMPLSNSCSVFVTRLLNQALIITLLIWLLSTLINVPYLLGTLLQLFQFVIPMSIITYCYFRILVNYILIGMVATFICCWLPLTAVNLVPLMAHVIAMSLVVWNPLLFFWLTRKQK |
| SC0019663 | -----------------------------------VCTVTAPMTQGFNIFLSTFSIAAIALDRFWEH---------------------TLSVLATQYAFPLFSLVFAYTRIA-------------------------------------------------- |
| SC0004604 | QKVPLHVSCSFNTPENTFLILLALKSRKGFSDLFLCLPKQYPIQAVNTFVSSLTLALIAMDRVLLTASIICYCVVWVVSIVIALPYSLTITVLAIQYLLPLPFALAYAYVQIG-------------------------YLFCHLVGISSACVNPITYALVNESFR |
| SC0005938 | ---------------------------------------------------------------------------------------------------------------------------LLNVHAIAMTSVVWNPLLYFWMS---- |
| MiV1ctg2742 | IFSFLYLIIWLAAILGNVSVLYVVSCSLAASDILMSMTSLPITQLGAVFVSSFTLTAIAVDRYILIKFLYTLAYIRLAIKFRTALSIVGILVLIVQFGLPVLISTLCYWQIGKIVSMFFQVAGLVLAWLPMNLINLWFAASHSIAMTSAVWNPIIYSFFNPQFR |
| MiV1ctg13 | FFAFLYSAIVLTSILGNILVILSVCVSLSVSDIVSIVSVGTITQGVSLCFSTFTLTLTSISVDRFLLICACRIVALNCAIALAISIPILF-------------------------IILAMVAVFFCCWAPSVIFNFLGTTTHCLSIRQVYN------------ |
| MiV1CTG2272 | -----YGCLFLLGLLGNGSVLLAFALNLILTDILLCTLAVPVTNSCAVFVTSWSLCAIAIDKFLHITAATITFVIWIISTLLNIPYLLGTGVMLFQFVLPMAIITYCYWRILILFSILLQVIAFIGCNTPITAVNLVPLIAHVIAMSAVIWNPFLFFWLTAKDK |
| MiV1ctg595 | LIFTAYLLVIAFGSIGNLLTIMAVLYRSVSEIFTIIYFLAPTTQGFNIFLSTFSITAIALDRLKLFLSLIFFSLIWLAS---------TVGVIILQYLLPLASIAFAYSRIA---------------------LNIFFTCSHLTAMCSACLNPCCYAFFNNNFR |
| MiV1ctg1852 | -------------------------TGRVEDLFTNSYSLAPTTQGFNIFLSTFSITAIALDRYQLKLSLIFFSLIWLAS-------------------LFLAADISPIFHDSQ---------------------EQLIYLLPHLTAMCSACLNPCCYAFFNNNFR |
| MiV1CTG549 | FFEFYFIRRRMTVAN----------LAISDLVILLCITALPITQGISVLISSYCLCLIAVDRYRSIHAQWLMVFWTFCIIISSPLFIVGISLLSIQFLIPTLIMTYCYWKIL---RVMLKVLIFMGSWMPLTFVNLLLLNVHAVAMTSVVSNPLLYFMSKVN- |
| EVF58827 | -------------------------CNLAVSDIFVTLTSLWLTQGASIFNSFTLTAIALDRYIVICFGIILAIWILSLLLCWLPYGF-------------------------LLKMLTLMVAIFGLLCWLPFNVLNILFLVSHLIAMSSSSSWNTVLYAWMNYHFN |
| EVF56136 | FFTAAYSIICGVGLLSNGLLIYVLFINLALSNFLLAALYIPFLPGSNIFCSTLTIAVMAVDRYYAVFAILVAGLIWVVALALSLPYLL--DEKRCTLVISGFSISGTVVDFGQSRSTPSAEIARSPQKPPTTTTNHAAICHGRQLMDAVRSPLALFTLDRRHR |
| EFV59568 | IFLICYLVIFIAGITGNVCVIISVAVSLSCSDIVVCLTSLPITQCMSTIISTFTLMAIAIDRFILIHATTMIVAIWTLAISISIPMLLGSIVLVLQFIIPLIVITFCYASISTNRMLISMVAVFVACWFFQVLLNVLSLIVHCIAMSSTLWNPILYAWLNDTFR |
| PPA05725 | IVIPLYILVFVLGLAGNLGLIVATLVNLGISDVILCLLSIPLTQAIGVFIGTFSLCAIAIDRFRLVYAIRITFLLWIISILVSTPYIYSIGILIIQFLVPFTIMAICYHSIFVTIILVSMVVIFGATALPHNVISIMATHFI--AMLSCVTNPILYAFLNPEFR |
| PPA10461 | -------------------------FSLSCSDLVVCSLSATIT---------------------------------------------GALLLTVQFVIPLTIIIISYSAISTNRMLIGMVVAFSASWIWSVAYNLCGIGTHCIAMTSTVWNPLVYALLNLQLR |
| PPA26426 | -------------------------------------------NSCSVFVTSWSLTAIAIDKFLHIIPMAIISYCYFRILNKYFFMVG--PISTSQLITKNSNRNGHNRTILVNYILIGMVMTFIGCWLPLTCVNLLPLIAHCCAMSLVIWNPLLFFWLTRKQK |
| PPA07498 | LLFSLYCLIFIVAVSGNVLVIFVVASNLALSDLIVNFTSLWLTQGMSIFISTLTLTAIAVDRYFVIRCLSIIVSIWVVSFSLVFPYAIGMIVMVLQAFYLPKVVSFKRYPA-------------------------------------------------- |
| PPA20680 | VNAQTGNRTRGFRLPGRSSTTELPVAFGNALTITVCTVTAPMTQAFNVFLSTFSIAAIALDRYVLILSLIFFIVWVVSILALPLLITLSVLITQYAFPLFSIVFAYSSIATHLLLLSVVVLFAVAWFPLNVFHVFFASCHVVAMCSACLNPLIYAFFNQNFR |
| PPA14003 | FYSIIFFLIFVLGLIGNG-LLISILINLAISDLLLCITSVPISQALSVLVSTYCLCFIALDRYTSIKAQCLMLLTWIIAAFFSSPLYIGFLLFIFQSLIPALIITFSYWQILMVYVLILMVVFMLSWLPLTVSMLLHCNVIRRMESPSLFLAIKVGWSIPSPP |

## E) Ghrelin-Obstatin/Neuromedin U receptors

| | |
|---|---|
| **nmur1** | KVTALYIFIFLVGVIGNTTCLVMKMNLAVSDLVTLCVGLPFEAETTSSVSILTILIFAIERYVAVNIGTIIGFTWIFSILCAMPFAIFHFSAIAFFALPLFTIVILYARIAINAILCAIVSAFFICYLPFQLQRLLYFISGFLFYLATIINPIAYNLASSRFR |
| **nmur2** | PTVIIYGTIFLLGLFGNICTCIVIAFSLAISDIIALILGLPMEIEFTSYASIMIICCFSFERWLAIRANVLIILAWTISFVCALPIAFIIFAFTVFFVIPAIVIMYAHIAVLKMLLSVVITFFICWLPFHIQRLLFYISGFCYYSNSAANPILYNILSQKYR |
| **nmur3** | ILTLIFVFVGLVGLIGNLLTVIVIFANLATSDFCLIVVGVSFDISLFTFASILTIVLLTAERFTAIRVKRFILLIWFVALLPSIFIGSFESAITITFVLPLFFIIYCFRILVIKMLVTVTAVFFVCYLPYHAQRLIYPIAGILQYISASLNPIFYNLMSVRFR |
| **nmur4** | TLAFVFSAISVIGVLGNLLVITVVLVSLAASDTLFFFASMPHEPYLAMNTSSLSILAFTIERYGIRATCIICGIWIFSMLYHSYWLFFLLDFVLWYVLPIMCDIIIYAKIGVVKMLAIVVAVFAVCWLPYRGMVVYINLSKTLVFINCAINPILYNLMSARFR |
| **npr20** | PVMIIYCLVFVVCLSGNFLTLIVMTSNLAIADLLVAIFCILQNTNIMPCTSAGILVLVSLEKYIAVHRWVATMIVWVISILVNFPYFLNSFSFVIWYAPVLPLILTALVFIYSRISVVRLLVAVVVSFALLTFPHHARLLYQPLSYIFLFISSAINPILYACLSKRFR |
| **npr21** | LLCVSYLFVFLATIIGNLIILLVFFANLTVADLFVGIFCVLQNLHFIPNVSAGILVLVSVERLIAVVLITSSAVVWLTSAVMNTPYVVATINFIVWYAVPLVILLCIYATIGVGRLAVGIVVAFAFFSLPRYVYFMWQPISFLLLFFNAAVDPFLYAFLSTRFR |
| CBG00234 | KVTALYIFIIGVFGNTATCLVMRLNLAVSDLVTLCVGLPFEAETTSSVSILTILVFAIERYVAVNMKSIIGLSWVFSILCATPFGIFHFSAIAFFVVPLLTILVLYARIAINAILCAIVSAFFICYLPFQFQRLLFYISGFLFYLATIINPIAYNVASSRFR |
| CBG06980 | PTVIIYGTIFLLGLFGNITCTCIVIAFSLAISDIIALILGLPMEIEFTSYASIMIICCFSFERWLAIRANVLIVLAWIISFICALPIAIIVFAFTVFFIIPAIAIVIMYAHIAVLKMLLSVVITFFICWLPFHIQRLLFYISGFCYYSNSAANPILYNLLSQKYR |
| CBG14471 | ILTGIFLFVGLVGLIGNLLTVIVISANLATSDFCLIIVGVTFDISLFTFASILTIVLLTAERFTAIRVKRFILLIWFVALLPSIFIGSFESAMAITFVLPLFFIIYCYFRILVIKMLVTVTGVFFVCYLPYHAQRFIYPIAGILQYISASLNPIFYNLMSVRFR |
| CBG03939 | TLAFIFSTISVIGVLGNLLVITVVLVSLAASDTLFFFASLPHEPYLAMNTSSLSILAFTIERYGIRATFIIAGIWAFSMIYHSYWLFFLLDFVLWYVLPILCDIIIYAKIGVVKMLAIVVAVFAVCWLPYRGMVVYINLSKTLVFINCAINPILYNLMSARFR |
| CBG03199 | PLMVIYCIVFVVCLVGNLLTLAVLTSNLAAADILVAVFCILQNTNVMPCTSAGILVLVSAEKYIAVNRKRACVAVWVISVGVNVPYFINIFSFVIWYLIPLICLTIIYSRISVVRLLVAVVVAFAVLTLPHHARILYQPLSYILLFMSSAINPILYACLSKRFR |
| CBG15954 | LLCVSYLFVFLATIIGNLIILFVFVANLTVADLFVGIFCVLQNLHFIPNVSAGILVLVSVERLIAVVLITSSAIVWITSAVMNTPYIYATINFIVWYAVPLVILLCIYATIGVGRLAVGIVVAFAFFSLPRYVYFMWQPISFLLLFFNAAVDPFLYAFLSTRFR |
| CJA00289 | KVSYHHMLGHEKAPNDENTCKVCKKMNLAVSDLVTLCVGLPFEAETTSSVSVLTILIFAIERYVAINIRTIIGFTWVFSILCAMPFAIFHFSAIAFFAIPLLTILILYARIAINAILCAIVSAFFICYLPFQLQRLLYFISGFLFYLATIINPIAYNLASSRFR |
| CJA03412 | IVRTEFCAFFIVPAIAGHIAWKLKSLKMLLSVVITFFICWLPFLYNILSQKGDHIANLISQGRRNTISVVSAGVEGLVKEYYDQLATFNGKPKCEPYFPESSGQSLTFGDITGIQCMCFIFGAFFCLIEAFKKQVAEASEKDKKEAASPPRPPVVRQPVPQSPR |

| | |
|---|---|
| CJA00933 | IFTLIFLFVGLVGLIGNLLTVIVIFANLATSDFFLIVIGVSFDISLFTFASILTIVLLTAERFQAIQVKIFILVIWIVALVPSIYIGFFETVMAITFVLPLFFIIYCYFRILVIKMLVTVTAVFFVCYLPYHAQRLIYPITGILQYISASLNPIFYNLMSVRFR |
| CJA12188 | TLAFIFSAISVIGVLGNLLVITVVLVSLAASDTLFFFASMPHEPYLAMNTSSLSILAFTIERYYGIRATCIIFGVWLFSMLYHSYWLFFLLDFVLWYVLPILCDIVIYVKIGVVKMLAIVVAVFAICWLPYRGMVVYINLSKTLVFINCAINPILYNLMSARFR |
| CJA06216 | PLMTIYITVFVVCLIGNLVTILVITANLAAADLLVAIFCILQNTNIMPCTSAGILVLVSVEKYIAVNRYRACFGVWFLSVTINFPFFLNTFCFVVWYMIPLISLVFIYSRISVVRLLVAVVVSFAVLTFPHHARLLFQPLSYILLFISSAINPILYACLSKRFR |
| CJA03720 | LLCVSYLFVFLATIIGNLIILLVFFANLTVADLFVGIFCVLQNLHFIPNVSAGVLVLVSVERLIAVVLVTSSAVVWLTSAVMNTPYVVATINFIVWYAVPLVILLCIYATIGVGRLAVGIVVAFAFFSLPRYVYFMWQPISFLLLFFNAAVDPFLYAFLSTRFR |
| SC0005743 | KVTVGYIVILLIGLLGNILAISVLR-----------------------------------------VHKVLCAAWLVSALCASPFGFFHLSALLFFVVPLSIIFVLHILIA-GIFSVSIVIALFICHVPFQIQRLS--------------------------- |
| SC0020418 | PTVIAYSLIFIIGTVGNICTCVVITFSLALSDLVVLFLGLPMEIEFTSYASILVICSFTVERWMAI---------------------------------------------------------------------------------------------------- |
| SC0014018 | LLSILFVLVGCVGLIGNMLTVLVIYASLAISDLLLIIVGVPFDISLFTFASILTIVSLTAERFVAI----------------------EKLLKIFILLRFFILVIPSLYIGLVPDFCRELNSQRGSCDYVTSQN---FTFASILTIVSLTAERFVAICYPFSHR |
| SC0025746 | SMAFVFATLAVVGIIGNILVITVVFVSLAFSDTLFFIATTPTEPYLAINSSALSITAFTVERFIGIRAKVIICGIWVFSFIYNFPWLFYVGDLVGFYVIPMLLNIVIYAKIAVVKMLALVVFVFATCWLPYRAMVVN--------------------------- |
| SC0005536 | PLIVIYLFVFSVCLLGNIFTIVVIVANLALADLLVAAFCILQNLHFIPCLSVGILVCVSIEKYLGE---------------------------------------------------------------------------------------------------- |
| MiV1ctg483 | PTAIIYGLILLIGLFGNICTCLVILFSLAVSDLLILTLGLPMEIEFTSYASILTITCFSVERWLAI----------------------------------------------SVVVMFFLCWLPFHIQRLLFYISGYCYYSNSACNPILYNILSAKYR |
| MiV1ctg461 | ----IFINLLLISVLLNFP-FQVIHASLAISDLLLILVGVPFDISWFTFNSILTIVSLTWERLVAI----------------------NFTQIWLIIIIWFISFFPSLFIGPSHNFSVMIAALFFVCYLPYHLERLIYHGTGLLQYISAALNPIIYNVMSRRFR |
| MiV1ctg604 | -MTVIYSLIALIGIIGNILVIVVVKVSLALSDCLFLCAAWPVEPYLGINASSMSIMAFTIERFIGIRAKLIILFIWCFCILYNSPWLYFVADFTTFYILPMFLYIYLYGKIAVIKMLALVAFIFAVCWLPYRAMVMYFCLKKTMIFFNCAINPVLYNLMSSRFR |
| MiV1ctg890 | -------------------------------------------LHLIPNSSAGILVYFFLIFIFLKSNACSTFNDICKEIKINKLFLISTVNLIVWYILPLTLLFSIYATIGVIRLCAVIVLAFALLSLPRYVNIFFQPTTFLLMFFASAINPILYAFLSQRFR |
| EFV53465 | TATLAYSTTALVGVFGNLLVIGVVLVSLAAADCLCFLSSTPTESFLAINASSLSIAAFTLERYIGIRAKMIICFIWIFSVLYTSPWLFFMTDMLLFYWIPFFLYIVVYGRIVIVNMLIAIVFTFTICWLPYRGLVVYLLVAKTMIYINCALNPILYHAMSAKFR |
| PPA17766 | QVTAAYLAILLIGVIGNAGTCLVIKLNLAVSDLVTLCVG--------------------------------------------------------------------------------------------------------------------- |
| PPA07900 | -------------------------------------------IEFTSYASVTVICAFSVERYFAIRAVTVILISWAVSFVAALPMAAIYVSFLIFFTLPAILMSFMYGHIAVIKVLVSVVVTFFAFWLPFHAQRLLFYLSGTCYYSNSVCNPILYNILSENYR |
| PPA26287 | -------------------------YEYMQHLNVINASG----EMIVRNCN-LSLVQDYAARFAML----------------------HVSSLFTLILTAIFALVGAVGAIG-----LTVWIVVGVPFDVLHAWRATFTFASTLTIVSLTFERYVAICHPFSLR |

## F) Somatostatin and galanin receptor

| | |
|---|---|
| **npr16** | LALTVTHLLLVALGSVNLIIILLIIIGLCLADFIYLADLILVAEATGKYASVLFVVLLAADRYIAMTAIFLSGLAWIAAFICSLPLYVISVCSVMIFILPGLVIFYCYYHVFVTRSVQRVVLFHLLCWSPFWLFNLFVNIIHLFPYVNCALNPVLYAYRAENFR |
| **npr17** | IMAVSYLLLFLLGTCGNVAVLTTIYIYVILVSCVDFGVCLSLPENFGKILSALILTAMSFDRYAGVITILLVLAVYAFITLCPLLWSFTIYQFILCYCTPLVLIAFFYTKLLISLYTLAVACFYFLCWTPFWMATLFMYFIHALPFTSAINWILYGALNGQLQ |
| **npr18** | LIAGFYALLFMFGTCGNAAILAVVHLTYICILSIVDFLSMLPIEHIGKIFSTFILVAFSIDRYCAVTVFVFLGIMFFVTCVMLSPILLTLYSFVLAYLMPLLFMIYFYYEMLIAIYTLAICSFHFICWTPYWISILYMYGVHALPYINSASNFILYGLLNRQLH |
| **npr24** | VAHLTWCLTALIGIPANLFVLAAIVFNLAVADLLFLCGIPVSLNAVSQFASAVFIAILSFDRYLAVAAFALSVAAWIMVILEMTPLFLISYTFALSYLIPLVAVWYFYFKIIVTIMGLAIVISYTHCWLPFWIVQWSTHFAFALQYINSAANPFLYVFLSDSFQ |
| **npr32** | FIIGTVGNGTVILMIINILTSMKRNFIYVLGLSIVDLLVILHLESVNKLLSSFLMTVLSWDRYMAVTALKVLLACTIFATVLLPVLYTLYTFVIGFAAPAFLIIIFYVQVIVTKRIVAVILFYFLCWTPQWTLNIMFFVAHLLVCFNSAANPVLYAYINRELR |
| **T02E9.1** | SICFVFGTLGNTAVLSYVFFITRSLVALCAVDLLVTVSIPFSLELSNKMCSTFILTALAFDRYMAIHTIYITTILATLSLFLISPVVLSAFLIFFAFLLPCTLLTYFYAKIVITIYTMAATFFYLSCHIPFWLPQIYTYYSHLLPFISAAFNWIFYARLNSQFK |
| CBG14230 | LALTVTHLLLVALGSVNLIVIYLIIIGLCLADFIYLTDLILVAEATGKYASVLFVVLLAADRYIAMTAIFLSGLAWIAAVICSLPLYIISVCSVMIFILPGLVIFYCYYHVFVTRSVQRVVLFHLLCWSPFWLFNLFVNIIHLFPYVNCALNPVLYAYRAENFR |
| CBG22950 | IMAVSYLLLFLLGTCGNVAVLTTIYIYVILVSCVDFGVCLSLPENFGKILSALILTAMSFDRYAGV----------------------TIYQFFLCYCTPLVLIAYFYTKLLISLYTLAVACFYFLCWTPFWMATLFMYFIHALPFTNSAVNWILYGALNGQLQ |
| CBG16052 | LIAGFYALLFMFGTCGNAAILAVVHLTYICILSIVDFLSMLPIEHIGKIFSTFILVAFSIDRYCAVFVFIFLTAMFVLTCVMLLPILITLYSFVLAYCLPLLLMIYFYYEMLIAVYTLAICLFHFACWTPYWISILYMYGVHALPYINSASNFILYGLLNRQLH |
| CBG01863 | VTWSLTALIGIPANLFVLAAIVYFRFNLALADLLFLCAIPVTFNAVSQFASAVFIAILSFDRYLAVAAVILSIAAWAMVIIEMTPLFLISYTFALSYLIPLVAVWFFYFKIIVTIMGLAIVISYTHCWLPFWIVQWSTHFAFALQYINSAANPFLYVFLSDSFQ |
| CBG00362 | FVIGTVGNGTVILMINILTSMKRNNIYVLGLSIVDLLVILHLPESVNKLLSSFLMTVLSWDRYMAVTAMKVLLACTLFATVLLPVLITLYTFVIGFAAPAFLIIIFYAQVISTKRIVAVILFYFLCWTPQWTLNIMFFVAHLLVCFNSAANPVLYAYINRELR |
| CBG23177 | SICFVFGTLGNTAVLSYVFFITRSLVALCAVDLLVTVSIPLSLELSNKMCSTFILTALAFDRYMAIHTIYITTILATLSFCLISPVVLSAFLIFFAFLLPCTLLTYFYAKIVITIYTMAATFFYLSCQIPFWIPQTYTYYTHLLPFISAAFNWIFYARLNSQFK |
| CJA01404 | LALTVTHLLLVALGSVNLIVILLIIIGLCLADFIYLADLILVAEATGKYASVLFVVLLAADRYIAMTAIFLSGLAWIAALICSLPLYVISVCSVMIFILPGLVIFYCYYHVFVTRSVQRVVLFHLLCWSPFWLFNLFVNIIHLFPYVNCALNPVLYAYRAENFR |
| CJA03613 | IMAVSYLLLFLLGTCGNVAVLTTIYIYVILVSCVDFGVCLSLP----------------------------------------------------------------------------------------------MYFIHALPFTNSAINWILYGALNGQLQ |
| CJA08149 | LIAGFYALLFMFGTCGNAAILAVVHLTYICILSIVDFLSMLPIEHIGKIFSTFILVAFSIDRYCAVTVFLFLGIMFLVTCGMLSPILLTLYSFVLAYLLPLVFMIYFYYEMLIAIYTLVICSFHFICWTPYWISILYMYGVHALPYINSASNFILYGLLNRQLH |
| CJA14285 | LTWSLTALIGIPANIFVLAAIVYFRFNLAVADLLFLCGIPVATNAVSQFASAVFIAILSFDRYLAVAAFVLSVAAWIMVILEMTPLFLISYTFALSYLIPLAAVWFFYFKIIVTIMGLAIVISYTHCWLPFWMVQWSTHFAFALQYINSAANPFLYVFLSDSFQ |
| CJA08780 | FVIGTVGNGTVILMINILTSMKRNSIYVLGLSIVDLLVILHLPESVNKLLSSFLMTVLSWDRYMAVTALKVLLACTIFATVLLPVLITLYTFVIGFAAPAFLIIIFYLQRAKPDGSSNRVKKVTKCWTPQWTLNIMFFVARIVAVILFYFLHLLFPGVLQLSG |
| XP 1894760.1 | -----------------------------------------------KYASVIFVVLLAADRYCAMIAVSTSVIAWIVAFSSALPLYLITFSSILIYAAPLALMTYFNYNVLVTRLVLCVVIFHAVCWSPFWLFNLLVNVIHLFPYINCALNPLLYAANAENFR |
| SC0008465 | ------------------------------------------------------------------SMRFGKCMKAEKGMQLSAANRQ------------------------VTRSVQRVVLFHLLCWLERIVVNII----HLFPYVNCALNPLLYAYRAENFR |
| SC0057342 | IMAVSYLLLFLLGTCGNVAVLTTIYIYVILVSCVDFGVCLSLP----------------------------------------------------------------------------------------------FWMFGTVPCKLHAVFENFGKILSALIL |
| SC0002879 | AIASFYALLFMLGTCGNAAILAVVHRKRNNTISRVICVIYR---------------------------------------------TLYSFLLHSSLAYLMPLLFMIYFYTVPLNPTQEQKFDHFRTPYWVSVLYMYGVHALPYINSASNFILYGLLNRQVR |
| SC0023371 | LVCCLAAVLGIPANLFVLIAIFYFRGNLAVADLLFLAGTPIVINGVSQFASAMFIAVLSLDRFLAVAAIAAVVFAWAIVILEITPLFM------------------------VTIMGLSIVISYTLCWLPYWLIQWSSYAAFALLYINRAVNPFIYVFLSESFK |
| SC0008331 | FVVGTIGNGVIVMINVLTSMRRTNTIYVLGLSFVDLVVILHLPESISKLLSSFIMTVLSWDRYLAVVALLVLLTCSLLAVILLLPVLITLYTFMIGFALPAILITLFYSRVKDPTSTRVQQVRKVTCWTPQWTLNIMFFVARIVAVILFYFLHLLVSF------ |
| MiV1ctg715 | ------------------------------------------------------------------VRCFKIYFIQLHILKISLCLADITASSILIFALPLAVIIFCYYHIFVTRLVLWVVIFHVLCWSPFWLFNLFVNIIHLFPYINCALNPILYVANAENFR |
| MiV1ctg1915 | -------------------------------------------ENINKLLSVAILTIMSFERFLAV--------------------------FHMICWGPFWLFVYVFFLRE---------------------------------------------------- |
| MiV1ctg1587 | -------------------------------------------------------------------------------------TPYWLLNIMSQFGFIVVSNYFQV---------------------------------------------------- |
| EVF55624 | --------------LGNIFVAIFILLNLSAADTIYLLFLPLVIESTTKYSSVYFIALLSVDRYLSITVLSTIIGWIMIASLMVPIYMNMIVGCYLICWTPYWVMNLTLMFSN---------------------------------------------------- |
| PPA27446 | -------------------------LFVAYTFALS-----------------------------------YLIPLIAIWIFYANIIQKM------------------------VSILGLAIVVSYTLCWAPFWSIQWANLTAYVLQYINSAANPFLYVFMSDAFK |
| PPA13927 | -------------------------IGLCLADFIYLTNLTLVAETTGKNYSVLFVVLLASDRYCAMTAIFLSVCAWIVAFLFSLPLYLISISSICLFAAPMVFIIYFYYHILVTRLVLWVVIFHVSLLAIQSLLFNI--------------------------- |
| PPA00294 | WIAAFYGMLFIIGTCGNVAILAIVHLTYLSVLCITDFLSMLPLEHISKIFSNFILVAFSVDRYLAVKVFLILGGVFLISCSLISPMIY---------------------------------------------------------------------------- |
| PPA09020 | -------------------------IYVLGLSIVDLLVLIHLPESVNKLLSSFLMCVLSWDRYLAV---------------------------------------------------------------------------------------------------- |
| **npr9** | IIPTIFAVIILVGLVGNALVVIVAFIGLAISDLMFLLLCVPFTQHTSAYCSVWTLTLMALDRYLAVNTVIALCFIYIIIIASQIPVGRFMTFNVFGYVLPLGISVVLYGLMLVTRLVLCVLITWALCWLPLNVCFFMQIASQVLAYTNSCLNPILYALMSQSFR |
| **npr15** | PYVCFFIILSVVGIIGNVIVIYAIALNLAVADLANLIFTIPEWECVFLFASISTQMIVCIERYIAINVLITVLVDWIFVACFASPYAVKLTEFLAFYFVPCFIITVVYTKVAVVKMLIACVAVYFVCYSPIQVIFLSILLMNALAMTCSASNPLLYTLFSQKFR |
| **Y54E2A.1** | LPGFLYLTVFLVGTIGNSMVIFVVNASLSTADLCLIWFCVPIMQQFTCFCSVLTMTMISFERFLAIRAKKVILLIWMSSAVLAVPTAVAMYQLLLLIIFPVLTMSICYARVSIVQMLISIVCMYTVCWLPTIVDELLRMGFNALTYCQSCINPILYAFISQNFR |
| CBG17369 | IIPSIFALIIMIGLIGNALVVIVATGLAISDLMFLLLCVPFTAQHTSAYCSVWTLTLMALDRFLAVNTMIALFILYTIIVCSQVHPRRLHDVQLIRIRSPLGISVILYGLMLVTRLVLCVLITWALCWLPLNVCFFMQIASQVLAYTNSCLNPILYALMSHSFR |
| CBG09746 | PFAVLFSILTVVGIIGNVFVIYAIALNLAVADLANLIFTIPEWECVFLFASISTQTIVCIERYIAINALITVAVSWIIVCCFAAPYAVKWSEFLSFYLSPCFIIIVVYTKVSVVKMLIACVAVYFLCYSPIQFMFLSILILNALAMTCSASNPLLYTLFSTKFR |
| CBG04262 | LPGFLYLTVFLVGTIGNSMVIFVVNASLSTADLCLIWFCVPIMQQFTCFCSVLTMTMISFERFLAIRAKKVILLIWMLSALLAVPTAVAMYQLLLLIIFPVLTMSICYARVSIVQMLISIVCMYTVCWLPTIIDELLRMGFNALTYCQSCINPILYAFISQNFR |
| CJA14394 | IIPTIFAVIICVGVIGNALVVIVATGLAISDLMFLLLCVPFTAQHTSAYCSVWTLALMALDRYLAVNTVIASAIIYAVIVMTQIPVGRFMTFNIFGYVLPLGISVVLYGLMLVTRLVLCVLITWALCWLPLNVCFFMQIASQVLAYTNSCLNPILYALMSQSFR |
| CJA01764 | PFAGLFVILSVVGIIGNIVVIYAIALNLAVADLANLIFTIPEWECVFLFASISTQMIVCIERYIAINALITVLVAWIFVACFASPYAVQAHRVPCLLLYPLPHHHWGVHKSCVVKMLIACVAVYFVCYSPIQVIFLSILLMNALAMTCSASNPLLYTLFSTKFR |
| CJA18843 | LPGFLYLTVFLVGTIGNSMVIFVVNASLSTADLCLIWFCVPIMQQFTCFCSVLTMTMISFESKLAR---------------------------------------------------------------------------------------------------- |
| SC0004612 | FGKANCINGSTHAVVLFMLVIFVVNASLSTADLCLIWFCVPIMQQFTCICSVLTMTMISFERWVRFRAKKVSKTSNILSLYGFCRRSWGMFQMLLLIVFPVVTMSICYARVSIVQMLISIVVMYTVCWLPTI-------------------------------- |
| MiV1ctg93 | IIPSIFALFALFGLVGNLLVVIVATGLTCSDLMFLTLCIPFTAQHSSAYFSVWTLTLMAADRFLAV---LKVGRIHGVYSYTFIMEQRFFSFNIFGYCLPLGITCVLYYLMLVTRLVLCVVIIWAVCWLPLNLCFFAQIFSQVVLAYCNSTLNPILYPLVSENF |
| MiV1ctg1567 | -------------NIFLVISILTYLMHTSVWCTRSRVLVIAFGPEKSIKIKKLIFSPKFLKKDLRVIYKWSEFLLTYALPIIIS-------VLLYSRICRVLWFKNFLKSAL---------------------------------------------------- |
| EFV54231 | ----IWYGLVLILGAIGNSLVITVVSSMSTADLLILLFCLPVQELMTMICSLMTMTTISIQRYAAVRALKTVLVTWLFSALLAVPILFKRFCPRREQSKKVTKMLVIILLLFKRKYSLAADSRTHNRRDSPISERSL--------------------------- |
| PPA20652 | YSPQYEKLYEESIAFENFLGVIMVAGLAVSDLMFLLICIPITaQHISAYMSVWTLSLMAFDRFLAI-----VAFLHGIYTYEFLIETRFCTFNFFGYVLPLAITVVFYAFML---------------------------------------------------- |

Supplementary Figure 2. Sequence of the Arthropod GPCR transmembrane (TM) domains used in for phylogenetic analysis. TM domains from non-model arthropods within each receptor family were extracted by sequence homology using the *D. melanogaster* TMs.  To facilitate visualization the TM1, 3, 5 and 7 were annotated in grey.

## A) Gastrin-cholecystokinin receptors

```
                 TM1                TM2         TM3                TM4                TM5                  TM6                      TM7
CCCKLR1    WVVPCYSAILLCAVVGNLLVVLTLVTNVFLLNLAISDILLGVFQAASVAVSSWTLVAISCERYYAIHANKIIAIIWLGSLVCMTPIAANLFLDLALLVLPLLALSFTYLFITVVKMLFVLVLEFFICWTPLYVINTMISFLQLLAYSSSCCNPITYCFMNASFR
CCKLR17    WLIPSYSMILLFAVLGNLLVISTLVTNVFLLNLAISDMLLGVLQAASVAVSSWTLVAISCERYYAIHAYKIIGFIWLGGILCMTPIAVNILLDFLLLVLPLLVLCVAYILITVVKMLFVLVLEFFICWTPLYVINTMISFLQLLAYSSSCCNPITYCFMNASFR
AGAP001022 LIIPLYAIIFLLSVVGNLLVILTLAVTNVYLLNLAISDLLLGVQAVSVSVAVWTLVAISLERYFAIHAYKMIGLVWTVSFLANSPLGYVLFHDVGLLFLPLLTMGFAYSMIVVIRMLFVIIVEFFVCWAPLHILNTVIALVQLMAYISSCCNPITYCFMNRRFR
AGAP001379 LLTVAYALVFILGLLGNISVVIVVKPTNRFIVNLAYADLLVNFQGVSVSASVNTLMAISIERCFAIRLIVTIIWFIALSINLPWLFVFFLFANLFFCYLPLIVISICYVIIWQVIKMVFVVIITFAISWLPLYAIFCLPVAQWLGASNSCINPILYAFMNRKFR
AAEL010207 LIIPLYAVIFLLSVVGNLLVILTLAVTNVYLLNLAISDLLLGVQAVSVSVAVWTLVAISLERYFAIHAYKMIALVWLLSFLVNSPLCYVLFLDAGLLCFPLITMGFAYSMIV----------------------------------------------------
AAEL017238 --IPLYSVIFLLAVIGNSLVILTLVITNLFLLNLAVSDLFLGVQASSVAVSAWTLVIISVERYYAIHAYRLIALIWCGSLFFMLPIAVNLFLDVILLVLPLLILAVTYSLIT----------------------------------------------------
GB18786    LIVPLYGIIFLLSIVGNSLVLITLAVTNVYLLNLAVSDLLLGVQAVSVSVGVWTLVAISLERYFAIHAYKMIAVVWTASLTWNAPILVNLFLDGTLLLVPLIVMSLAYSLIAVIRMLFVIVLEFFVCWAPLHVINTWISLVQLLAYVSSCCNPITYCFMNRKFR
ISCW005570 LRITLYSIIFVFAVVGNVLVLVTLVVTNVFLVNLAVSDLLLGVQEGYGRQCTRTTGVNDEAY-----------------------------------------------------------------------------------------------------------
ISCW009627 IRILLYAIIFVCAVVGNSLVILTLVVTNVFLLNLAVSDLLLGVQVASRLALPTTCLHRARFFTPLP-------------------------------------------------------------------------------------------------------
ISCW005948 -------------------------------------------PAVSVCVSAWTLMAMSVERYFAIHAQRTIAAVWVASLLLMLPIALTLFLDALLLVLPLLGMTATYSRIAIIRMLFAVVVEFFVCWTPLYVLNTVISFLQLLAYASSCCNPITYCFMNRTFR
```

## B) Neurokinin/ neuropeptide FF/ orexin receptors

```
NKD        IWAIIFGLMMFVAIAGNGIVLWIVTTNYFLLNLSIADLLMSSLANVTVSTSVFTLVAISFDRYIAIKVRIILVLIWALSCVLSAPCLLNLIILVLTYGIPMIVMLICYSLMGVVRMFIAIVSIFAICWLPYHLFFIYYLGFYWLAMSNAMVNPLIYYWMNKRFR
DTKR       LWSILFGGMVIVATGGNLIVVWIVMTNYFIVNLSIADAMVSSLAMLSICASVFTLMAISIDRYVAICNLAIAAVIWLASTLISCPMMINILIIILTYFLPIVSMTVTYSRVGVVKMMIVVVLIFAICWLPFHSYFIIYLAIYWLAMSNSMYNPIIYCWMNSRFR
Lkr        LLSIFYGGISIVAVIGNTLVIWVVA-NMYIANLAFADVIIGLFQALSVNVSVFTLTAIAIDRHRAIVSKFIIGGIWMLALLFAVPFAIRYTLVFVQYLVPFCVISFVYIQMAVIKMLIIVVIIFGLCWLPLQLYNILWFCCDWLAMSNSCYNPFIYGIYNEKFK
CG10823    VYCVAYIVVFLVGLIGNSFVIAVVLTNYFIVNLAIADILVIVFQGVSVAASVYSLIAVSLDRFIAIRARIMIIGIWVIALVTTIPWLLLLANLVACYLLPMSLITLCYVLIWVIKMLVAVVILFVLSWLPLYVIFARMPVAQWLGSSNSCINPILYSVNKKYRR
CG30340    WKICTFLPLIAFGLYGNFSMVYVIATNLIIANMAVADLLTLAI-------AVLNLSVVSYDRLTAI-VQIVVVCTWVSGILLASP---WYVLITILVWLPLGIMLICYIAIFVAKTLFIVVVVFAALRLPFTILVVLWYISQYLMFLNAAVNPLIYGFNNENFR
AGAP002824 AWGVVFGAMLLVAITGNCIVLWIVLTNYFLLNLSVADLLMSSLANMSVASSVFTLVAISFDRYIAIKARIFLLIIWALSCVLAAPCLMNLVFLILTYGIPMLIMIVCYSLMGVVRMFIIVVTIFAICWLPYHLFFVYYLGFYWLAMSNAMVNPIIYYWMNGRFR
AGAP001592 SSPPTLVAGPYPEPNGTAEIGELNARFGFTVNLSIADAMVSTLAILSICASVFTLMAIAIDRYVAIATLCVAASIWIVGTIISCPSLLNIVFMLLTYFLPIGSMTYTYARVGVVKMMMIVVIIFAVCWLPFQIYFILYLAIYWLAMSNSMYNPIIYCWMNLRFR
AGAP011179 VKLCVFLPIIVFGLLGNGILLEVIFSHLLIANLVVTDFLTLLVEGGLLVTSVLGMCVISYDRLSAVGTVAAIVFCWLMGFSIAMPLSLWEFLLVMLVWVPLGVMLVAYTLIITLYIVLLAFIVVRVPFTIMVFVYYNLDHFQVVELTNIVRNFNEMHFFSTTV-
AGAP010851 LLSLFYGTISILAVIGNSLVIWIVLTNMFIANLALADVTIGVFQLISVNVSVFTLTAIAVDRHRAIISKFVISTIWMLSFALAAPILFRYVLVLVQYFVPLFVISFVYIQMAVIKMLIIVVALFGVCWFPLQLYNILWFVCDWLAMSNSCYNPFIYGIYNEKFK
AAEL006947 ------------------------------VNLSIADAMVSTLAILSICASVFTLMAISIDRYMAIATLCIAAAIWIVGIIISSPMLLNVIFMFLTYFLPIGSMSFTYARVGVVKMMMVVVVIFAVCWLPFQIYFIAYLGIYWLAMSNSMYNPIIYCWMNSRFR
AAEL008267 LKLSVLIPIVLFGILGNVSLLEIIFTHMLIANLALIDLVTLLIEGGLLITSILALCVVSYDRLAAISVIVASIGCWILGFVAALPLAIWDYIIILLVWLPLSVMMVTYSTILTLFIVLITFIIVRIPFTVMILIYYKWWFAKVAFIFLYSAMNPIIY-------
AAEL008282 VKAATFLPLITFGIFGNIALLCIIFTNMLIANLAVADLATLIILGSLLITSVLCLCGISYDRLTAIGVTVIIACAWLAGFLLALPLTIWHVLIGALVWFPLSVMICCYSLIFCLFVVLIVFIILRIPFTTLVFIRYYWYVSHYLMFLNAAINPLIY--------
AAEL011026 LLSIFYGTISIIAVIGNSLVIWIVLTNMFIANLALADVTIAVF---------------------------------------------RYILTLVQYFVPLCVISFVYIQMAVIKMLIIVVALFGICWFPLQLYNILWFVCDWLAMSNSCYNPFIYGIYNEKFK
AAEL006636 LLSIFYGTISIIAVIGNSLVIWIVLTNMFIANLALADVTIAVFQLLSVNVSVFTLTAIAVDRHRAIISKFVISAIWMMSFALAAPTLFRYILTLVQYFVPLCVISFVYIQMAVIKMLIIVVALFGICWFPLQLYNILWFVCDWLAMSNSCYNPFIYGIYNEKFK
GB13925    IWTLLFAGMIIVATGGNLIVIWIVMTNYFLVNLSIADAMVSTLAVLTICASVFTLMAISIDRYMAIATICIAIVIWIVGAVLSLPMLLNVIFMVLTYVLPIGSMTFTYARIGVSS-------------------------------------------------
GB30014    IWTLLFAGMIIVATGGNLIVIWIVMTNYFLVNLSIADAMVSTLAVLTICASVFTLMAISIDRYMAIATICIAIVIWIVGAVLSLPMLLNVIFMVLTYVLPIGSMTFTYARIG----------------------------------------------------
GB18532    ---------------------------------------------------------------MAIATICIAIVIWIVGAVLSLPMLLNVIFMVLTYVLPIGSMTFTYARIGVVKMMIVVVLIFAICWLPFHVYFIIFLGIYWLAMSNSMYNPIIYCWMNSRFR
GB15294    VKIISILPIIIIGSFANGGLIYVILTNLLIVNMCIADLGTCLI--ALTLVAVFNLSTISYDRVSAIMMYILLFSTWIFGISVAIPLIYWHIFAGLSVWAPLTIMAICYTAILILALIVLAFIICRVPFTALIIRRAQWYISRYLVLVNAAINPLLYGCSSSSLR
GB11188    LLSILYGTISFLAVVGNSLVIWIISTNFFIANLALADIVIGLFQVLSVNVSVFTLTAIAIDRHRAITAKIIIAGIWLLAGSLATPMAISGLLVFLQYLTPLSIISCVYARMAVIKMLIIVVTLFAICWLPLQMYNVFFFFFDWLAMSNSCYNPFIYGIYNEKFR
GB10679    VLIASHSIVFVIGLIGNALVCIAVYTNYFIVNLAVADFLVLLL--VSVTVSILTLTFISIDRWYAIWAKNAIIGIWAIALLFDIPDLVIIVKLIFLYTGPLIFMSVAYWQIVAAKMLVTVVVIFAICYFPVHLLSVLSLIAHGLCYFNSAVNPLIYNFMSGKFR
BMGA008039 SWICVFSIMLIIAVGGNAIVIWIVITNYFLVNLSLADLMMSALANVTVAASVFTLTGISFDRFQAICSLIAIGGIWLGGMVLATPYLLQIAFFIVTYAVPMVGMSFFYTAMGVVKMFILVIVIFGICWFPYHGYFIYYLGFYWLAMSNAMVNPIIYYWMNAK--
BMGA002506 --------MVVVATVGNLVVIWIVLTNYFLVNLSVADAMVSTLAVLSISASVFTLLAISVDRYVAIATLGITAAIWAWSSFISSPNLINVLFMVLTYFMPIIAMTYAYSRVGVVKMMIVVVVIFAVCWLPFHVYFVVYLGIYWLAMSNSMYNPIIYCWMNSKFR
BMGA008933 IKSSMMILVGVSGIFLNCVILTILISNCLIGNMALIDFLLLFFQASLLLASVGAVMLVSYDRLAAAAAPKVIVITWIVSSALALPWTIWHLLLTMLVWVPLGIMIITFKRRNRVVKNILHINKDSIEFSIKIDEMTT---------------------------
BMGA000313 -----------------------------------------------------------MKEFKARVAKVIIVFIWLLAFTLAAPMEMRLLLYIFQYVIPLCVITFAYVHMAVIKMLVLVVALFALCWLPLQSYLLLFFCFDWLAMSNSCYNPFIYAIYNDGQS
ISCW022739 LLSLFYGLISLVAVAGNFMVMWIVATNFFIANLAVADIIIGLFQVLSVNVSIFTLTAIALDRYRAVRAKFIICAIWTLALAAALPYALNHCLVCLQYFFPLLTICFVYARMGVRCSH-----------------------------------------------
ISCW015326 LLSFCYGTISLVAVLGNAFVLWIVATNYLIANLAISDIIIGLFQVVSVNVSIFTLTAIAVDRYRAITARALISVIWAVSMLAAVPYALNNALVCLQYFVPLLVICYVYEKIR----------------------------------------------------
ISCW022222 LLSFCYGLISLVAVAGNSMVLWIVATNFFIANLAVADIVIGLFQVLSVNVSIFTLTAIALDRYRAVNAKVLISIVWAVSTAAALPYALNHVLVGLQYFLPLCVICYTYGRIG----------------------------------------------------
ISCW022730 LLSFCYGLISLVSIVGNILVLWIVATNIFIANLAVADIIIGVFQVVSVNVSVFTLTAIAIERYRAITAKILILIIWISSLVVACPNAVNHALVCVQYLLPLTIVCYTYGRIWVIKMMAIVVIIFALCWLPYQTYNLLWFCSHWLAMSNSCYNPFIYAIYSEKFS
```

## C) Neuropeptide Y receptors

```
NepYr       IVYMLYIPIFIFALIGNGTVCYIVYTNYFIASLAIGDILMSFFQAVSVLVSAYTLVAISIDRYIAIYATFIIAGVWFIALATALPIPITLSLFALQFVVPLGVLIFTYARITMVKMMLTVVIVFTCCWLPFNILQLLWFAFHWLAMSHCCYNPIIYCMNARFR
CG32547     LFLIQYAALALLGVVLNVIIVVYIMTHAFIINLALCHFVQCALQDIPLHVAMISHILIAWDRMRWLPGFVCCCATWLTGMVIALPYPITRGLFLLMYCGPAILLSYLYIRTSKQRNFGSMAATQVVCMCPLMILRFAYLMFVWVAFLPTVIFPCIYASQILPRD
NPFR1       MLISMYGVLIVFGALGNTLVVIAVIRNLFILNLAISDLLLCLVQALCIFVSTISITAIAFDRYQVIGAVTILAGIWALALLLASPLFVSIFSLCVQYLVPILIVSVAYFGIYTNCLLISIAIIFGVSWLPLNFFNLYYAICHMIGMSSACSNPLLYGWLNDNFR
SNPFR1      FFYVLYATVFVLGVFGNVLVCYVVLTNIFITNLALSDILLCVLQGCSIYISTLTLTSIAIDRYFVITCIGIIVSIWVIALLATVPYGMGAITTTLQFVLPFFIISICYVWISTNRMLIAMVAVFGLSWLPINVVNIFFFVAHSIAMSSTCYNPFLYAWLNENFR
AGAP000351  TSYFLYITIFVTAVIGNSIVLFIVQTNFFITNLAVGDLMMTLFQAVSVLVSAYTLVAISGDRYIAICSKCLIGIVWIIALITAVPIAITIALLTTQFIVPLVVLIFTYTRIAMIKMMVTVVIVFTICWLPFN----FWFAFHWLAMSHSCYNPIIYCMNARFR
AGAP000115  VVWLAYTAILAASLVGNCSVLAIVATNLFLANLALGDLLMTLFQAVSVLVSAYTLVALSADRYRAIIALLLIGLVWVGAAATALPIPLSLTLALAQFALPLGTLVYTYACIGTVRMTLAVVAAFVVCWLPFNALLLAWFACHWLAMSHCCLNPLIYCMNAKFR
AGAP000383  LFILLYAIPTALGLTLNVMIIVYISTHAFLVNLAVCHCVQCAFQDIPLHVAMLSHLLIAWDRKRWL-AFVCSCASWLAGLVIALPYPIMRGLFVIMYCAPMTLLGYLYIRTSTQRHLGAMASSQVLCVCPLMILRLAFLMFVWIAFLPTIIVPWIYAAWVLSKP
AGAP004123  ILIIMYGTLIVFGATGNSLVVLAVARNMFIVNLAVSDLLLCLVQATSIFVSTISITAIALDRYQVIGAIAILTGIWIISIVLASPMFISAFTLCVQYVLPILIVSMAYLRIYTNYLLISIALIFGVSWLPLNLFNLF---------------------------
AGAP012378  VFCVLYSSIFVLGVFGNVLVCYVVFTNLFITNLALSDILLCVLQGCSVYISTLTLTSIAIDRFFVITCITIIVLIWSFAIMVTMPYGLSIVTSILQFVLPFIIMAFCYICVSTNRMLISMVAIFGISWLPLNVVNMCFFIAHLTAMSSTCYNPFLYAWLNDNFR
AAEL017005  TSYFLYVLIFVTAVIGNSIVLFIVQTNYFITNLAVGDLMMTLFQAVSVLVSAYTLVAISGDRYIAICSKVLIGVVWVIALITAAPIAITTTLLMTQFVIPLLVLIYTYTRIA----------------------------------------------------
AAEL008296  LIYLAYSTIFLTSIVGNVAVFLVVHTNLFIANLALGDMLMTIFQAISVLVSAYTMIAISADRYLAILARILILLVWTGALATAAPIPVSLTLMTLQFLAPLIVLITTYVQIAMIKMMITVVAVFTICWLPFN----IWFLFHWLAMSHSCYNPIIYCMNEKFR
AAEL015418  PDLPGVQRDLPDLDRRNVAVFLVVHTNLFIANLALGDMLMTIFQAISVLVSAYTMIAISADRYLAILARILIFLVWTGALATAAPIPVSLTLMTLQFLAPLIVLITTYVQIAMIKMMITVVAVFTICWLPFN----IWFLFHWLAMSHSCYNPIIYCMNEKFR
AAEL012190  ------------------------------------------QDIPLHVAMISHLLIAWDRKRWL-AFVCSCASWLAGMVIALPYPIMRGLFLIMYCGPMTLLGYLYIRTSTQRHLGAMASTQILCVCPLMILRLAYLMFVWMAFLPTVIVPWIYANWVLSRP
AAEL010626  ILIVLYSILIIFGATGNSLVVMAVARNMFIVNLAVSDLLLCLVQATSIFVSTISITAIALDRYQVIGAIVILTGIWIISLVLASPMFISVFTLCVQYLLPILIVSIAYLRIYTNYLLISIALIFGISWLPLNLFNLFYAICHMMGMSSACSNPLLYGWLNDNFR
AAEL013505  VFCLLYSSIFILGIFGNVLVCYVVFTNLFITNLALSDILLCVLQGCSVYISTLTLTSIAIDRFFVITCITIIVLIWIFSMLVTLPYGLSIATSILQFVLPFIIMAICYICVSTNRMLIAMVAIFGISWLPLNLVNMSFFIAHLIAMSSTCYNPFLYAWLNDNFR
GB13527     IIYFVYSTVFVVALTGNGLVCYVVHTNFFIVNLALGDILIALFQAVSVLVSAYTLVAISIDRYIAIQAQLLILAVWMLAMLISLPIAISIALLVLQYVIPITVLVFTYTSIAMVKMMVTVVIVFTVCWLPFNILNLIWMILHWLAMSHSCYNPVIYCWMNARFR
GB19597     ---------MALAVTANILVIAVVFTNYFVVNLSVADLLVTTIQGVAVAASVFTITAMSIDRYLAISTVFVIIALWVVALIIFSPLLRSIYVHFLDFYHAGFVVVLSYIMMGIAWILLLLAVLFALCWLPYNVLMLLNNAISYCLFLGHANSALNPTVYCFMTR
GB30377     IFYILYVHIFMLGVFGNVLVCFVVATNLFITNLALSDVLLCVLQGVSVYISTLTLTSIAIDRFMVIVCLLVIIGIWIFALLLTSPYGISSITSILQFVLPFFVIAFCYICVSTNRMLIAMVTVFGISWLPLNIVNVIFFMTHCLAMSSTCYNPFLYAWLNDNFR
BMGA002442  ------------------------------------------QAVSVLVSAYTLLAISIDRYMAIAAKMVVAGVWGGAIATATPIYVTCALLVLQFVLPLSALVCTYARIAMIKMMVIVVAVFTVCWLPL----------------------------------
BMGA002458  ------------------------------------------QAVSVLVSAYTLLAISIDRYIVIAAKMVVAAVWGGAIITAAPIPITFALLTLQFTLPLTALVYTYGRIAVLWTLHEGDEDWAVWPGMP------WFASHWLAMSHSCYNPLIYCYMNAKYR
BMGA000016  -------------------------RNMFIVNLAVSDALVCVVQAISIFVSTISITAIALDRYQLIGALVTMFFIWVTAFILASPLYISLLSLIFQYLLPVLVVVMAHIQIHTPAILIAIAVTYVISWLPLNVFNLVYAVCHMFGMSSAVSNPLLYGWLNDNFR
BMGA012112  -------------------------KSLLQLTRAAAFTTTCITWSVRVKLTALSLTARAAHGELPL-HVIIVAGVGPRERRDAVDVGDSKKKLQPSLLGPQPQTSTLRSRR-LGNVLMGIAGIFAMCWCPHAAIVIC----HYALLLGYAHSALNAVAYWVLNR
BMGA012041  AFCILYTVIFVLGVFGNVLVCYVVFTNLFITNLALSDILLCVFQGTSVYISTLTLTSIAIDRFFVITCIFIVIFIWVFSLVVTCPYGLGVFTTVLQFLIPFLVIAVCYTCVSTNRMLISMVAIFGISWLPLNLINIFFFLAHSMAMASTCYNPFLYAWLNENFR
BMGA012114  AFCVAYTIIFAVGIFGNALVCYAVITNLFITNLALSDILLCVFQGCSVYISTLTLTSIAIDRFFVITCLGLIIFIWFFALLVTFPYGYGAITTIMQFVIPFIVMAFCYTCVSTNRMLIAMVAIFGLSWLPLNLINMSFFLAHFIAMSSTCYNPFLYAWLNENFR
ISCW020603  LMYIMYIAVSVAAIGGNGIVCYIVLTNMFIMNLAIGDILMASLQCVTVFISAYTLIAISVDRYTAIRSKIIIGVVWLVALVTPLPTALSMALMILQYFFPLLALIFTYTRIA----------------------------------------------------
ISCW020600  ------------------------------------------AVTALTCSVLTLSAISCDRFTAIRTGGVLASVWLISAAVASPLLMTGVCVARSASKQLYYTAVTVALFFVFVMSSAYALILHALWRER---------------HPGEANANNTLLYTRAKR
ISCW000923  FFYCIYVLIFVTGICGNILVCFVVFTNIFIANLALSDILLCALQGVSVYISAFTLMAIAIDRFFVVVCFTIIVSVWVSSALLTLPYGISSLTTTLQFLVPLSIVTFCYVRVCTNRMLISMVLIFAVSWMPLNLYNLVFFLSHAIAMSSTCYNPFLYAWLNENFR
ISCW000924  -------------------------TNFFITNLGLSDILLCTLQGVSVYISSFTLMAIAIDRFFVIVCFMIIVCIWLTGALLTLPYGISFSTSTLQFVVPFGIISFCYMRVCTNRMLISMVVIFGASWLPLNLYNLVFFLSHAVAMSSTCYNPFLYTWLNENFR
```

## D) Bombesin receptors

```
CCHa1r      IVPILFALIFVVGVLGNGTLIVVFLPNTYILSLALADLLVIITKDVSIGVSVFTLTALSGDRYFAIMTLATAVSIWLLAILCGLPALIVLLHFLVYYAIPLVVIAVFYVLIAVAVTVLAFVVIFGICFLPYHVFFLWRIVAYCMSFANSCANPVALYFVSGAFR
CCHa2r      IVTVLYTLIFIVGVLGNGTLVIIFFPNTYILSLALADLLVILVKDISIGVSVFTLTALSGERYCAILTVFTAVMIWILAILLGMPSVLVAGKALVYYLLPLSIIGALYIMMAVARMVVAFVVVFFICFFPYHVFELWRIVGFCTSFLNSCVNPVALYCVSGVFR
AGAP003631  IVPVVFALIFIVGIVGNGTLVVIFLPNTYIFSLALADLLVILIEEISIGVSVFTLTALSADRYCAILTVIVAFLIWVLAIICAIPSAVTVLNSIVYYFLPLSIIGILYVLMAVARMVITFVIVFIVCFLPHHVVELWRITGFCLSFLNSCINPVALYCVSGVFR
AGAP011452  IVPILFAAIFIIGVLGNGTLIIVFLPNTYILSLALADLLLIVTKDVSIGVSVFTLVALSGDRFFAIMTITTAVLIWLLAIAFAVPAIVVLGKFLVYYAVPLFIIGIFYALIAVAITVLAFVVIFGICFLPSHLFMLW---------------------------
AAEL012385  -------------------------FVHYIFSLALADLLVIFVREISIGVSVFTLTALAADRYCAIVTVIVAVLIWLLAIAFAIPSATTIINSVIYYFLPLAIIGILYILMAVARMVITFVVVFIICFLPHHVFQLWRITGFLLSFLNSCINPVALYCVSGVFR
AAEL017410  -------------------------------------------------------------------------------------------TIINSVIYYFLPLAIIGILYILMAVARMVITFVVVFIICFLPHHVFQLWRITGFLL--------------------
AAEL003473  IVPILFAAIFIVGVLGNGTLIIVFLPNTYILSLALADLLLIVTKDVSIGVSVFTLTALSGDRFFAIMTITIAVLIWILAIIFAIPAILVLGKFLAYYAVPLCIIAIFYALIAVAVTVLAFVVIFGICFLPSHLFMLW---------------------------
GB16092     IVPIVFLLILLIGLTGNGVLALTILPNIYVFSLALGDLLVIMTKDISIGVSVFTLTALSADRFFAIFTVIVAALIWLLAIMCAIP-GSLVCRFFIYYAVPLIIIAFFYILMAVAKMVMAFVIVFAVCFFPQHVFMLWRILGFCLAFTNSCINPIALYCVSSTFR
GB10022     IVPVVFLLILVVGVIGNGILVFTLVPNTYVLSLALGDLLVIITKDISIGVSVFTLTALSAERYCAILTILTACLIWVLAIVLAMPAAFVMFKFLAYYAIPLLVITGFYLGMAVGKMVIAFVVIFFICFLPHHVFMLWRILGFCLSFINSCVNPIALYFISGTFR
BMGA008802  LVPILFAVIFVVGVLGNGTLVIVYAPNTYIFSLALADLLVILIKDVSIGVSVFTLTALSAERYCAILPLVCATFIWGAAFIFAIPAAITIAKALIYYGLPLIVITVFYSLMAARKSVACMVLIFVIAK----------------------------------
BMGA009039  IVPILFALIFVIGVVGNGTLVAVFVPNTYILSLALADLLVIITKDVSIGVSVFTLTALSADRYFAIVNVTNEILRTIFANIFRVLTAK-----------------------------------------------------------------------------
BMGA009040  VLSTQNMPGEMQGTQRQITRVGSERHSVLFCALATADMLNATP---------------------------------------------------------------------VAVTVLAFVLVFAACFLPSHVFMMWRIVGFCLSFLNSCVNPIALYCTSGIFR
ISCW015075  VVPTLFALIFIVGLLGNGTLILVFIPNIYIMSLSIGDFIVIAGRDVSIGVTVLTLTVLSIDRYVAIVTILLAISVWIVAILMAIPGTHVLLKFLIQYAIPLVIIGTFYCLMAVAKIALAFVLLFAVCFFPNHVFMMWKIMGYVMTFVNSCLNPVALYLVSGVFR
```

## E) Ghrelin-Obstatin/Neuromedin U receptors

```
PK2r1      TLSVGYALIFIAGVLGNLITCIVISTNFYLFNLAISDMILLCSSETAANATVLTITAFTVERYIAIRAVKFIFAIWIAALLLALPQAIFAVSGFLFFGGPMTAICVLYVLIGVIRMLVAVAVAFFICWAPFHAQRLMDYTSGVLYFLSTCINPLLYNIMSHKFR
PK2r2      ALTVCYALIFVAGVLGNLITCIVISTNFYLFNLAVSDLILLVSSEMAANATVLTITAFTVERYIAIRAIKFIFAIWLAAFLLALPQAMFAVSGFIFFGGPMTAICVLYVLIGVIRMLVAVAVAFFLCWAPFHAQRLMDYTSGVLYFLSTCINPLLYNIMSHKFR
PK1r       PVTVVYSLIFITGVVGNISTCIVIKTNYYLFSLAISDFLLLLSAETSANATVLTITAFTVERYIAIRAIRIIVLVWIMAIVTAIPQAAFQLSTFIFFLAPMSIILVLYLLIGVLRMLVAVVVCFFLCWAPFHAQRLITYVSGVLYYLSTCINPLLYNIMSHKFR
capaR      LITIIFGGIFITGVVGNLLVCIVIITNYYLFSLAVSDLLYLLFSEACTYVSVFTIVAFSMERFLAIRAIRIITALWIVSFISAIPFGLFEVSFCIFFVIPMILIILLYGRMGVIRMLAAVVITFFVCWFPFHLQRLIFSIAGFAYYVSCTVNPIVYSVMSRRYR
CG34381    IFITLYTLVFCCCFFGNLLVILVVTTNFFLANLAFADFCVGLFHSLSYTASIFILVVICMERYFAIRLRMVIVTVWITSAVYSTPKFVDMINFVLLYVMPLLVMTVLYSKIAMHNSMYHHHPHHHHHHHQHHQLQSASGGSSTTSLSRKQSSKYEKRGVSITES
AGAP003244 QVTVLFVGILVTGVIGNLIVCLVIVTNYYLFSLAVSDLIFLLLSEASTYVSVLTIVAFSMERFLAIRPVRIIAGLWVVSLLSAVPFAVWELSTCLFFAFPMLIMVVLYGRMGIIRMLVFPCLNTPSTFNANSSFFVV---------------------------
AGAP002881 TSMVFCIIIMCLGVIGNVMVPIVILTNIFLTNLSIADLLVLLVELTVAHASVLTILAISFERYYAIRALLICLAAWTVAAILTS----FVGSIVLFFIVPLLILVVLYSVIAVIFMLGAVVVSFFVCLLPFRALTLWLYFCRIMLYMNSAINPILYNLMSSKFR
AGAP000658 PITIIYLLIFITGVVGNISTCIVIATNYYLFSLAVSDFLLLVSAEMSANATVLTITAFTIERYFAIRAVRFICVIWLIAIVSAIPQALFELSTFLFFFAPMTMITILYALIGVLKMLVAVVVAFFICWAPFHAQRLVTYISGILYYLSTCINPLLYNIMSNKFR
AGAP003076 PITIFYCFIFVAGIVGNLSICVVIATNYYLFNLAVSDFLLLLFSETAANATVLTITSFTVERYIAIRAIKFVIVIWLVAFGLATPQALFEVSSFLFFVGPMTLIAVLYVLIGVIRMLVAVVATFFICWAPFHAQRLMNYTSGILYFLSTCINPLLYNIMSHKFR
AAEL017335 -VTILFTGILITGVVGNLIVCLVIITNYYLFSLAVSDLILLLLSEASTYVSVLTIVAFSMERFLAIRPVRIIAGLWIVSLFSAVPFAVWELSTCLFFAGPMVVMIVLYGRMG-----AAVVITFFVCWAPFHAQRLLFSVAGWLYYVSCTVNPILYNVMSHRYR
AAEL012796 PMTIIYVLIFITGVIGNISTCIVISTNYYLFSLAISDFLLLISAETSANATVLTITAFTIERYVAIRAIRFIFAIWLVAIVSAVPQALFELSTFLFFFAPMTLITVLYALIGVLKMLVAVVVAFFICWAPFHAQRLVTYISGILYYLSTCINPLLYNIMSNKFR
AAEL003747 PITIFYCFIFVVGIVGNLAICIVIATNYYLFNLAVSDFLLLLFSETATNATVLTITSFTVERYIAIRAIKFVIAIWLVAFGLATPQALFEVSSFLFFVGPMTVIAVLYVLIGVIRMLVAVVATFFFCWAPFHAQRLMTYISGILYFLSTCINPLLYHIMSHKFR
AAEL017048 PISIIYSTIFVIGVLGNVITCFVISTNYYLFSLAISDLLLLLTAETSANATVLTITAFTVERYVAIRAVRYILAIWMIAMCLAVPQALFTISTVIVFVFPMCVLTILYILIGNGAQYLAVVIAFFLCWAPFHAQRLFTHISGILYFVSTCINPVLYNIMSHKFR
GB18327    PITVIYAVIFVTGLVGNVSTCVVIATNYYLFSLAVSDLLLLISAETSANATVLTITAFTVERYIAIRAVKFVIVIWLLALCLAVPQAI-EISTMLFFVLPMTIIIVLYILIA-------------------------LTATVNRNHLSGGTNHWDSGRRKSAAQ
GB11169    PITLTYVVIFVTGFVGNIITCIVIWTNYYLFNLAVSDLLFLILSETSSYVSVLTIVAFSIERYLAIRAIRSIFGAWLIALIFAMPFATYQLSCILFFLIPMVFIAVLYIRIGITRMLSAVVITFFICWAPFHVQRLLYPLTGCLYYFSTTINPILYNVMSAKYR
GB12896    PLTIIYMIIFVTGIFGNITTCTVIITNYYLFSLAISDLILLVL---------------------------------------------YELSTIIFFLIPMLIILVVYTRMGVIKMLSAVVILFFICWAPFHTQRLLYILSGCLYYFSTTINPILYNLMSIKYR
GB18762    PVTIIYVSIFVTGIIGNISTCIVIATNYYLFSLAVSDLLLLASAETSTNASVLTITAFTVERYLAIRAVKLILVVWLVALSFALPQALFELSTFLFFVVPMSLITVLYALIGVLKMLVAVVIAFFICWAPFHVQRLITYISGVFYYISTTINPILYNIMSNKFR
GB13260    TSMITCIIVMVLGIIGNLMVPIVVFTNIFLVNLSVADLCVLLIELTVAHASVLTILAISFERYYAIRATFLCFVAWIVAALCTSPILLILFTIIVFFVIPFLILVVLYTVIAVMLMLGTVVLCFFLCLLPFRALTLWLYFCRVMLYLNSAINPILYNLMSTKFR
BMGA002245 ----------------------------------------------------------------------------------------------------------------VILMLGTVVLSFFICLLPFKALTLWLYFCRVMLYLNSAINPILYNLMSSKFR
BMGA010612 ----------------------------------------------------------------------------------------------------------------VIKMLVAVVVAFFICWAPFHAQRLVTYISGIFYYMSTCINPILYHIMSNKFR
ISCW012018 -------------------------------------------AEAPSYASVLTIVAFTVERYVAIRAVRIIAIIW------------------------------------------------------------------------------------------
ISCW022759 PMTFIYSILLVSGVIGNVCTCIVIATNYYLFSLAVSDLLLLVLSETSTNASILTITAFTIERYVAIRAVKFVVVIWVLSALCAIPLAV-ELSTFAFFLLPMSVILVLYVCIADVDHRCPVVHNGKSATAAEKEMVQP---------------------------
```

## F) Somatostatin and galanin receptors

```
Star1      FTVVLYGFVCIIGLFGNTLVIYVVLTNIYILNLAVADECFLIGTSITSFTSSIFLLIMSADRYIAVIAKVVSAIAWSTSAVLMLPVILILYTFFLGFATPLCFILSFYYLVIVTRLVLTVISVYILCWLPHWISQVAFLLLGALVYSNSAVNPILYAFLSENFR
AlCR2      ITMILYALVCIIGLFGNTLVIYVVMTNIYILNLAIADECFLIGTSITSFTSSIFLLIMSADRYIAVVSKLVSAFAWMTSVLLMLPVILILYSLVLGFATPLTFILVFYCLVIVTKLVLTVISAYIFCWLPHWISQVAFLACGCLSYSNSAMNPILYAFLSDNFK
AGAP010486 LFMFLYAVVCLVGVLGNTLVIYVVLTNMYILNLAIADECFLIGTSVTQFTSSIFLFIMSADRYIAIVSRIVSLLAWLASALIMLPIMITLYSLILGFVIPLCFIMTFYCLVIVTKLVLTVITVYILCWLPYWISQVAFLLVGCLGYINSAINPILYAYLSENFK
AAEL012920 VFMILYAVVCLVGVMGNTLVIYVVLTNMYILNLAIADECFLIGTSITQFTSSIFLFIMSADRYIAIVSRVVSALAWLASALIMLPIMITLYSLILGFVVPLCFIMTFYCLVIVTKLVLTVITVYILCWLPYWISQVAFLLVGCLGYINSAINPILYAYLSENFK
AAEL012356 ISMILYAVVGLIGLFGNTLVIYVVLTNMYILNLAIADQCFLIGTSITQFTSSIFLFIMSADRYIAVVSKIVSFFAWTTSALIMLPVMLTLYSLILGFAVPLSLILMFYYLVIVTKLVLTVITVYVLCWLPYWISQVAFVLVSCLGYSNSAMNPILYAFLSDNFK
GB20155    VNQILYSIVCIVGLLGNTLVIYVVLTNMYIVNLAIADECFLIGTSINQFTSSIFLFIMSADRYIAVISKVVSLTAWATSALFMIPIFLTLYTFILGFAIPLILILIFYFLVIVTKLVLTVITVYVLCWLPYWLMQVAFLLAGFLSYSNSAMNPILYAFLSDNFK
BMGA006675 VTQVLYALVCIVGLLGNTLVIYVVLTNMYIVNLAIADECFLIGTGINQFTSSIFLCIMSADRYIAVVSRIVSAAAWTASAAIMTPIFMTLYSFALGFAAPLTLIFIFYCLVIVTKLVLTVIAVYVLCWLPYWAFQMAFLVAACFSYSNSAMNPILYAFLSDNFK
ISCW007666 FSKLLYGLVCLVGLCGNSLVLYVVLTNMYIFNLALADEMFLVGTSINQFTSSLLLTVMSADRYVAVIAKFVCLTAWTVSALLMVPVYMTLYSFTLGFAIPLLLILLFYVLVIVTYLVLTVITVYVICWLPYWAGQLYFLLSQLLSYANSAVNPILYAFLSDNFK
DAR1       IVPVFFGIIGFAGLLGNGLVILVVVTNLLIINLAVSDILFVIFIVVTCHCSVYTLVLMSFDRFLAVNATLAIMCAWITIVTTAIPVALQVSFFLSSYVAPLTLICFLYMGMLVTRMVVVVVLAFAICWLPIHVILVLQIISHVVAYTNSCINPILYAFLSDNFR
DAR2       IVGFFFGVIAITGFFGNLLVILVVVTNLMIVNLAAADLMFVILIVVTAFASIYTLVLMSIDRFLAVITLIAIVTLWIVVLVVSVPVAFQVTFFISSYLLPLMIISGLYMRMIVTRLVVVVVIAFASLWLPVQLILLLQVTAQTLAYSSSCINPLLYAFLSENFR
AGAP012268 MVPVFFGFIGLAGLLGNGLVVLVVATNLLIINLAAADLLFVVFIVVTAHASAYTLVLMSLDRFLAVNASLAICLTWFVILTTAIPVAI------------------------VTRMVVVVVLVFAFCWCPIQVILLLQIVSHVLAYTNSCINPVLYAFLSDNFR
AGAP003658 MVPVFFGFIGLAGLLGNGLVVLVVATNLLIINLAAADLLFVVFIVVTAHASAYTLVLMSLDRFLAVNASLAICLTWFVILTTAIPVAI------------------------VTRMVVVVVLVFAFCWCPIQVILLLQIVSHVLAYTNSCINPVLYAFLSDNFR
AGAP001773 IVAIFFGFIGITGLLGNALVVLVVLTNLLIINLAIADLLFVVFIVVTAHASIYTLVLMSLDRFLAVNTVLAITILWVLIITTGLPVSFHIAFFLSSYVVPLILISVLYMWMLVTRLVVIVVAAFASLWFPIQVILVLQIVSHVLAYTSSCINPLLYAFLSENFR
AAEL007169 MVPVFFGLIGLAGLIGNGLVVLVVATNLLIINLAAADLLFVIFIVVTAHASAYTLVLMSLDRFLAVNAVIAITVAWIVIVTTAIPVAIHVSFFLSSYVIPLTLISMLYVGMLVTRMVVVVVLAFAICWCPIQIILVLQIVAHVLAYTNSCINPVLYAFLSDNFR
AAEL006076 IVAIFFGLIGITGLLGNALVVLVVLTNLLIINLAVADLLFVIFIVVTAHASIYTLVLMSLDRFLAVNTLWAITILWVLIITTALPVLF---------------------------------------------------------------------------
GB19021    VVPIFFGMIGILGLVGNSLVVIVVATNILIINLAVADLLFVILIIVTAYASVYTLVLMSLDRYLAVHAIIAICIAWAMIFAISTPAFFQMSFFLMSYLLPLMLICFFYICMLVTRLVFVVVGVFAFCWCPIQVILVTQIASHILAYTNSCVNPILYAFLSDSFR
Q8WPA2     VVPIFFGFIGIVGLVGNALVVLVVATNLLIINLAVADLLFVIFIVVTAHASVYTLVLMSLDRFMAVNALLAIACIWVVILTTAIPVGIQMSFFLSSYVIPLALISVLYMCMLVTRMVVVVVVVFAVCWCPIQIILLVQIVSHVLAYMNSCVNPVLYAFLSENFR
ISCW014938 VVPILFGAIAVVGFFGNALVVLVVLTNILIINLAMADLLFIVFVIVCAYASIYTLVLMSFDRFLAVNAYLAILLMWVVILLACIPVLFQLCFFMTSFLVPLALVFILYVLMLVTRLVVVVVVVFAVCWCPVQVVLVLQIASQVLAYTNSCVNPFLYAFLSENFR
ISCW016382 VVPVLFGAIAIVGFFGNALVVLVVLTNILIINLAMADLLFIVFVIVCAYASIYTLVLMSFDRFLAVNAYLAIFLTWVMILLACIPALFQICFFMSSFVVPLALVFILYVLMLVTRLVVVVVVVFAVCWCPVQIVLVLQIASQILAYTNSCVNPFLYAFLSENFR
ISCW016381 VVPILFGAIAVIGFFGNALVVLVVLTNILIINLAMADLLFIVFVIVCASASIYTLVLMSFDRFLAVNAFLAIFMTWIVILLACIPALFQICFFMASFVIPLTLIFILYVLMLVTRLVVVVVLVFAVCWCPVQVVLVLQIASQILAYTNSCVNPFLYAFLSDNFR
ISCW001334 VVPILFGAIAVVGFFGNALVVLVVLTNILIINLAMADLLFIVFVIVCAYASIYTLVLMSFDRFLAVNAYIAIFLTWVVILLACVPALFQICFFMFSFVVPLALIFILYVLMLVTRLVVVVVVVFAVCWCPVQIVLVLQIASQILAYTNSCVNPFLYAFLSENFR
```
